# Disparate forms of heterogeneities and interactions among them drive channel decorrelation in the dentate gyrus: Degeneracy and dominance

**DOI:** 10.1002/hipo.23035

**Published:** 2018-12-07

**Authors:** Poonam Mishra, Rishikesh Narayanan

**Affiliations:** ^1^ Cellular Neurophysiology Laboratory, Molecular Biophysics Unit Indian Institute of Science Bangalore India

**Keywords:** adult neurogenesis, computational model, degeneracy, hippocampus, parametric variability, sparse connectivity

## Abstract

The ability of a neuronal population to effectuate channel decorrelation, which is one form of response decorrelation, has been identified as an essential prelude to efficient neural encoding. To what extent are diverse forms of local and afferent heterogeneities essential in accomplishing channel decorrelation in the dentate gyrus (DG)? Here, we incrementally incorporated four distinct forms of biological heterogeneities into conductance‐based network models of the DG and systematically delineate their relative contributions to channel decorrelation. First, to effectively incorporate intrinsic heterogeneities, we built physiologically validated heterogeneous populations of granule (GC) and basket cells (BC) through independent stochastic search algorithms spanning exhaustive parametric spaces. These stochastic search algorithms, which were independently constrained by experimentally determined ion channels and by neurophysiological signatures, revealed cellular‐scale degeneracy in the DG. Specifically, in GC and BC populations, disparate parametric combinations yielded similar physiological signatures, with underlying parameters exhibiting significant variability and weak pair‐wise correlations. Second, we introduced synaptic heterogeneities through randomization of local synaptic strengths. Third, in including adult neurogenesis, we subjected the valid model populations to randomized structural plasticity and matched neuronal excitability to electrophysiological data. We assessed networks comprising different combinations of these three local heterogeneities with identical or heterogeneous afferent inputs from the entorhinal cortex. We found that the three forms of local heterogeneities were independently and synergistically capable of mediating significant channel decorrelation when the network was driven by identical afferent inputs. However, when we incorporated afferent heterogeneities into the network to account for the divergence in DG afferent connectivity, the impact of all three forms of local heterogeneities was significantly suppressed by the dominant role of afferent heterogeneities in mediating channel decorrelation. Our results unveil a unique convergence of cellular‐ and network‐scale degeneracy in the emergence of channel decorrelation in the DG, whereby disparate forms of local and afferent heterogeneities could synergistically drive input discriminability.

## 1 INTRODUCTION

The ability of a neuronal population to effectuate channel decorrelation has been identified as an essential prelude to efficient neural encoding, as this form of response decorrelation ensures that information conveyed by different neuronal channels is complementary (Chow, Wick, & Riecke, 2012; Padmanabhan & Urban, 2010; Pitkow & Meister, 2012; Tetzlaff, Helias, Einevoll, & Diesmann, 2012; Wiechert, Judkewitz, Riecke, & Friedrich, 2010). The critical importance of local circuit heterogeneities—including those in intrinsic properties, in synaptic strengths and in neuronal structure, observed either under baseline conditions or achieved specifically through adult neurogenesis—in achieving such response decorrelation has been recognized across different brain regions (Aimone et al., 2014; Aimone, Deng, & Gage, 2010; Aimone, Deng, & Gage, 2011; Chow et al., 2012; Coulter & Carlson, 2007; Dieni, Nietz, Panichi, Wadiche, & Overstreet‐Wadiche, 2013; Edgerton & Jaeger, 2011; Goard & Dan, 2009; Lledo & Valley, 2016; Luo, Axel, & Abbott, 2010; Marin‐Burgin, Mongiat, Pardi, & Schinder, 2012; Padmanabhan & Urban, 2010; Padmanabhan & Urban, 2014; Pitkow & Meister, 2012; Severa, Parekh, James, & Aimone, 2017; Tetzlaff et al., 2012; Wang, Scott, & Wojtowicz, 2000; Wiechert et al., 2010). Studies in the olfactory bulb (OB), one of the two prominent brain regions that express adult neurogenesis, have assessed the impact of these local heterogeneities on response decorrelation (Luo et al., 2010; Padmanabhan & Urban, 2010; Padmanabhan & Urban, 2014; Wiechert et al., 2010), emphasizing the critical importance of intrinsic heterogeneities and lateral inhibition in the emergence of response decorrelation. However, despite the dentate gyrus (DG) being the other prominent brain region expressing adult neurogenesis and despite the widespread literature on the role of DG in pattern separation (Aimone et al., 2010; Aimone et al., 2011; Aimone et al., 2014; Deng, Aimone, & Gage, 2010; Kropff, Yang, & Schinder, 2015; Leutgeb, Leutgeb, Moser, & Moser, 2007; Yassa & Stark, 2011), it is surprising that the impact of distinct forms of local and afferent heterogeneities on channel decorrelation has not been assessed in the DG.

In the DG network, there are at least four distinct forms of heterogeneities that could mediate response decorrelation (the first three are local to the DG network, whereas the fourth is afferent onto the network): (i) heterogeneity in intrinsic ion channel and excitability properties of the neurons; (ii) nonuniformities in the local synaptic connectivity; (iii) structural heterogeneities in neurons introduced by adult neurogenesis; and (iv) input‐driven heterogeneity that is reflective of the distinct sets of afferent inputs that impinge on different neurons (as a consequence of the unique divergence in DG connectivity). Which of these distinct forms of heterogeneities play a critical role in mediating channel decorrelation in the DG when they coexpress? What is the impact of cell‐to‐cell variability in ion channel properties and excitability on channel decorrelation in the DG network receiving different patterns of inputs? Is there a relative dominance among these disparate forms of heterogeneities when they *coexpress*? How does the contribution of local network heterogeneities to channel decorrelation change in the presence of unique, sparse, and orthogonal external inputs, an important and unique form of afferent heterogeneity that expresses in the DG network (Aimone et al., 2011; Aimone, Wiles, & Gage, 2006; Aimone, Wiles, & Gage, 2009; Li et al., 2017)?

In this study, we systematically and incrementally incorporate the four different forms of heterogeneities into conductance‐based network models of the DG and delineate the impact of each form of heterogeneity on channel decorrelation. Specifically, we used a stochastic search algorithm spanning an exhaustive parametric space (involving experimentally determined ion channel and neurophysiological properties) to reveal cellular‐scale degeneracy in the DG, whereby disparate combinations of passive and active properties yielded analogous cellular physiology of excitatory granule (GC) and inhibitory basket cell (BC) populations. Next, we further expanded the parametric search space to encompass biologically observed heterogeneities in local/afferent network connectivity and in neurogenesis‐induced alteration to neuronal structure and excitability. We systematically assessed channel decorrelation in different DG networks, each built with incremental addition of the four distinct forms of heterogeneities. We found that in the absence of afferent heterogeneities, that is, when the DG network was driven by identical afferent inputs, the three forms of local heterogeneities were independently and synergistically capable of mediating significant channel decorrelation. Under these scenarios where the network received identical inputs, we demonstrate a hierarchy of heterogeneities—synaptic, intrinsic, neurogenesis‐induced structural, in increasing order of dominance when they coexpress—in effectuating channel decorrelation. Importantly, when we incorporated afferent heterogeneities into the network to account for the unique activity‐dependent sparseness and neurogenesis‐driven synapse formation in DG afferent connectivity (Aimone et al., 2006; Aimone et al., 2009; Aimone et al., 2011; Li et al., 2017), we found that the impact of all three forms of local heterogeneities were suppressed by the dominant role played by afferent heterogeneities in mediating the emergence of channel decorrelation. These conclusions point to degeneracy (Edelman & Gally, 2001; Rathour & Narayanan, 2017), specifically with reference to the emergence of channel decorrelation, with the relative contributions of individual forms of heterogeneities critically regulated by several factors including the degree of divergence of afferent inputs. In elucidating a dominance hierarchy among disparate forms of heterogeneities in terms of their ability to mediate response decorrelation, our results quantitatively demonstrate that the ability of local heterogeneities to decorrelate identical inputs does not necessarily translate to them being effective in decorrelation when different degrees of afferent heterogeneities are present.

## 2 METHODS

**Figure 1:**
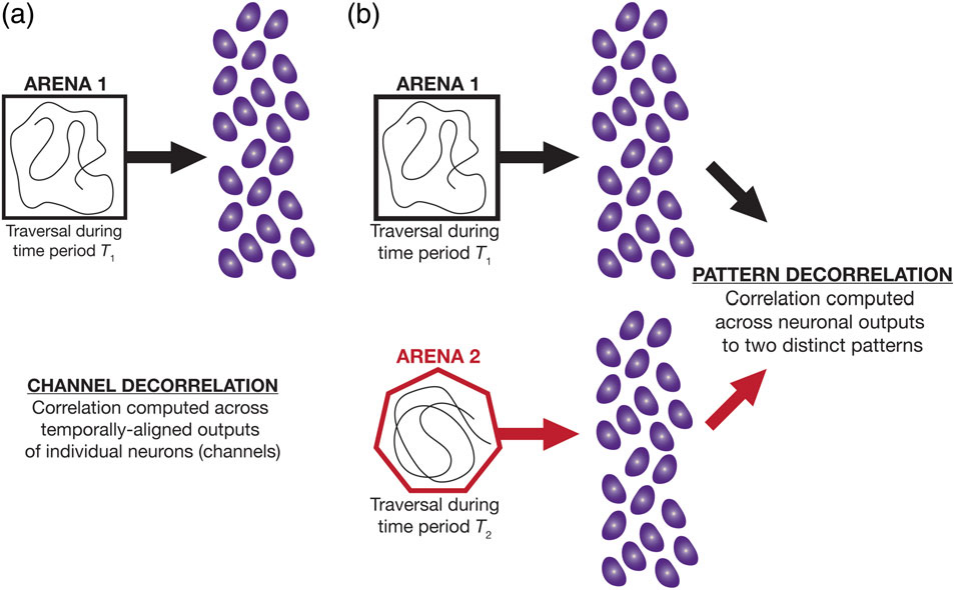
Two forms of response decorrelation: channel decorrelation and pattern decorrelation. (a) Illustration of channel decorrelation. A trajectory of an animal in Arena 1 results in temporally aligned inputs arriving onto a network of neurons. Individual neurons within the network elicit outputs to these inputs. Channel decorrelation is assessed by computing pair‐wise correlations across temporally aligned outputs of individual neurons (channels) within the network, when inputs corresponding to a single pattern (Arena 1) arrive onto the network. Channel decorrelation is computed to determine redundancy in individual neuronal outputs to afferent inputs. (b) Illustration of pattern decorrelation. Two trajectories of an animal in two distinct arenas (Arena 1 and Arena 2) results in distinct sets of inputs arriving onto the *same* network, at two different time periods *T*_1_ (Arena 1 traversal) and *T*_2_ (Arena 2 traversal). Neurons in the network elicit *two sets of outputs* (as opposed to the single set of outputs analyzed with reference to channel decorrelation) as the animal traverses Arena 1 or Arena 2. Pattern decorrelation is assessed by computing correlations across these two sets of neuronal outputs when inputs corresponding to two different arenas (patterns) arrive onto the same network. Pattern decorrelation is computed to determine the ability of neuronal outputs to distinguish between the two input patterns (in this case, corresponding to the two arenas). In this study, our focus is on assessing the impact of distinct biological heterogeneities on channel decorrelation [Color figure can be viewed at wileyonlinelibrary.com]

The principal goal of this study was to systematically assess the impact of different forms of heterogeneities on response decorrelation in the DG. Our specific focus in this study is on channel decorrelation (Figure 1a) (one form of response decorrelation that is distinct from pattern decorrelation; Figure 1b), where we assess the correlation between response profiles of individual channels (neurons) to afferent stimuli. Specifically, channel decorrelation decreases the overlap between channel responses, resulting in a code that is efficient because the information conveyed by different channels is largely complementary (Wiechert et al., 2010). In assessing the role of different forms of heterogeneities on channel decorrelation (Figure 1a), we took advantage of the versatility of conductance‐based neuronal network models, and distinguished between four different types of heterogeneities: (i) *intrinsic heterogeneity*, where the GC and BC model neurons that were used to construct the network had widely variable intrinsic parametric combinations yielding physiological measurements that matched their experimental counterparts. These heterogeneous model populations were obtained using independent stochastic search procedures for GCs and BCs; (ii) *synaptic heterogeneity*, where the synaptic strength of the local GC–BC network was variable with excitatory and inhibitory synaptic permeability values picked from uniform random distributions; (iii) *neurogenesis‐induced heterogeneity in age/structure* of the neuron, where the DG network could be made entirely of mature or immature neurons, or be constructed from neurons that represented different randomized neuronal ages; and (iv) *input‐driven or afferent heterogeneity*, where all neurons in the GC and BC populations received either *identical* inputs (absence of afferent heterogeneity) from the EC, or each GC and BC received unique inputs (presence of afferent heterogeneity) from the EC. The presence of afferent heterogeneity is representative of the sparseness of afferent connections from the EC to the DG, whereby neurons in the DG do not receive the same set of EC inputs during an arena traversal. We present the methodology to account for four different forms of heterogeneities, also providing details on the construction of the network, the measurements, and the analysis techniques used.

### 2.1 Intrinsic heterogeneity: Multi‐parametric multi‐objective stochastic search

**Figure 2:**
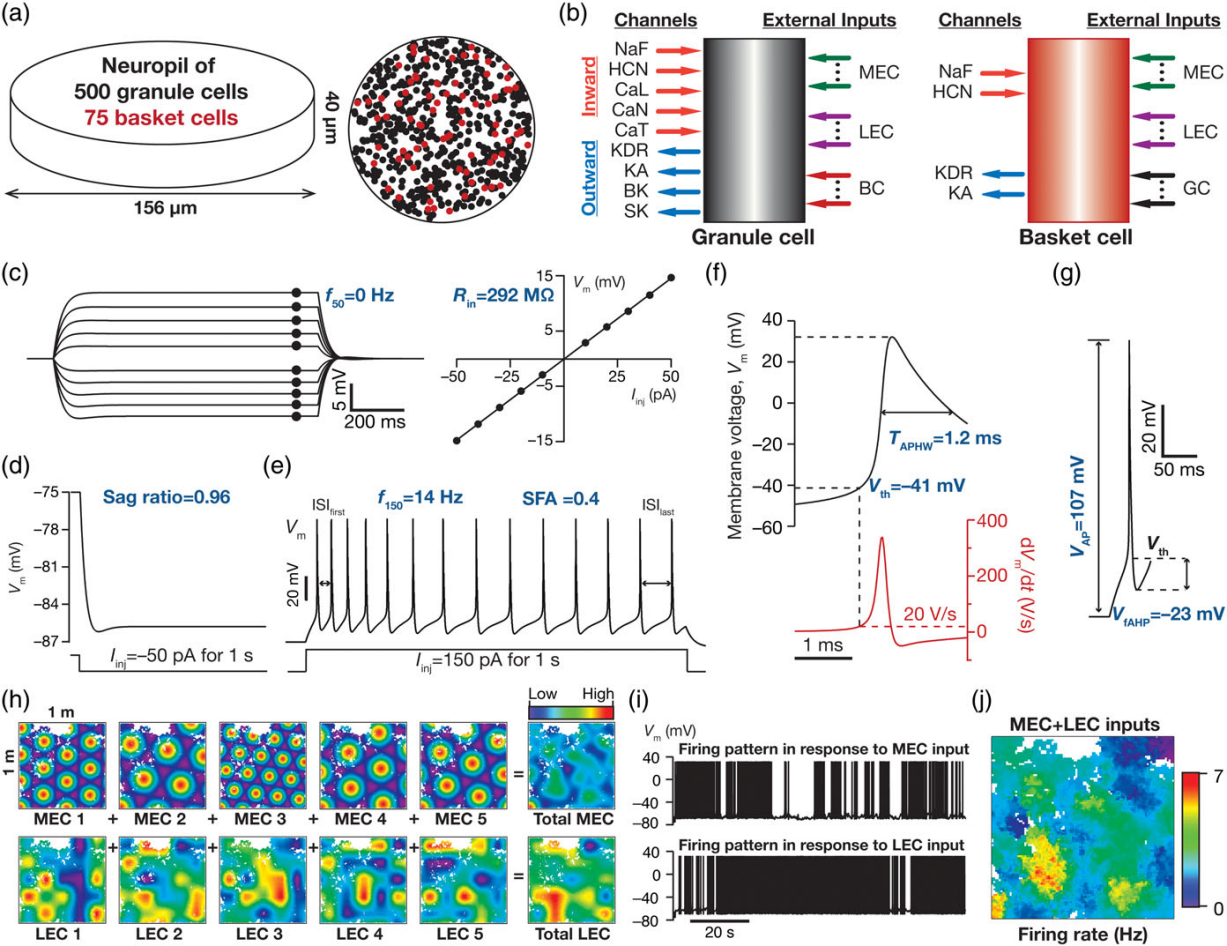
Model components and measurements. (a) Schematic representation of the cylindrical neuropil of 156 μm diameter and 40 μm height (left) with the top view (right) showing the distribution of 500 GCs (black) and 75 BCs (red). (b) Conductance‐based models of GCs (left) and BCs (right) expressed different sets of ion channels and received external inputs from several MEC and LEC cells. (c–g) The nine physiological measurements used in defining the GC populations: input resistance, *R*_in_, measured as the slope of a *V–I* curve obtained by plotting steady‐state voltage responses to current pulses of amplitude −50 to 50 pA, in steps of 10 pA, for 500 ms (c); sag ratio, measured as the ratio between the steady‐state voltage response and the peak voltage response to a −50 pA current pulse for 1 s (d); firing rate in response to 50 pA, *f*_50_ (c) and 150 pA current injection, *f*_150_ (e); spike frequency adaptation (SFA) computed as the ratio between the first (ISI_first_) and the last (ISI_last_) interspike intervals in spiking response to a 150 pA current injection (e); action potential half‐width, *T*_APHW_ (f); action potential threshold, computed as the voltage at the time point where *dV*_m_/*dt* crosses 20 V/s (f); action potential amplitude, *V*_AP_ (g) and the fast after hyperpolarization potential (*V*_AHP_). (h) Inputs from MEC (top) were modeled as grid structures with randomized scale and orientation, whereas inputs from LEC (bottom), carrying contextual information, were represented as smoothed and randomized matrices comprised of active and inactive boxes. Schematic color‐coded representations of individual inputs (5 MEC and 5 LEC cells) and their summations (separate for MEC and LEC inputs) are superimposed on the virtual animal trajectory in an arena of size 1 m × 1 m. (i) Sample GC voltage trace in response to total MEC (top) and LEC (bottom) current inputs. (j) Color‐coded rate map obtained by superimposing firing rate output from an isolated GC in response to both MEC and LEC inputs, as the virtual animal traverses the arena [Color figure can be viewed at wileyonlinelibrary.com]

The well‐established stochastic search strategy spanning multiple model parameters that satisfied multiple constraints on physiological measurements (Anirudhan & Narayanan, 2015; Foster, Ungar, & Schwaber, 1993; Goldman, Golowasch, Marder, & Abbott, 2001; Mittal & Narayanan, 2018; Mukunda & Narayanan, 2017; Prinz, Bucher, & Marder, 2004; Rathour & Narayanan, 2012; Rathour & Narayanan, 2014; Srikanth & Narayanan, 2015), an approach that we refer to as multi‐parametric multi‐objective stochastic search (MPMOSS), provided us an ideal route to generate a heterogeneous population of GC and BC neuronal models. The choice of this strategy ensured that we have models that are constructed with disparate parameters, but matched with their experimental counterparts in terms of several physiological measurements. In performing MPMOSS on granule cell model parameters, we first tuned a base model that matched with nine different active and passive physiological measurements of granule cells (Figure 2c–g). The passive model parameters of granule cell were as follows: the resting membrane potential (*V*_RMP_), −75 mV; specific membrane resistance, *R*_m_ = 38 kΩ cm^2^; and specific membrane capacitance, *C*_m_ = 1 μF/cm^2^. This allowed us to set the passive charging time constant (*R*_m_ *C*_m_) to be 38 ms (Schmidt‐Hieber, Jonas, & Bischofberger, 2007). Then, to set the passive input resistance (*R*_in_) of the cell to match the experimental value of 309 ± 14 MΩ (Chen, 2004), we set the geometry of the model cell to be a cylinder of 63 μm diameter and 63 μm length (*R*_in_ = *R*_m_/(πdL) = 38 × 10^3^ × 10^−2^ × 10^−2^/(π × 63 × 10^−6^ × 63 × 10^−6^) = 305 MΩ).

We introduced nine different active conductances into the GC neuronal model (Santhakumar, Aradi, & Soltesz, 2005): hyperpolarization‐activated cyclic nucleotide gated (HCN or *h*), *A*‐type potassium (KA), fast sodium (NaF), delayed‐rectifier potassium (KDR), small conductance (SK), and big conductance calcium‐activated potassium (BK), *L*‐type calcium (CaL), *N*‐type calcium (CaN), and *T*‐type calcium (CaT). The channel kinetics and their voltage‐dependent properties were adopted from experimental measurements from the GC (Aradi & Holmes, 1999; Beck, Ficker, & Heinemann, 1992; Ferrante, Migliore, & Ascoli, 2009; Magee, 1998). The reversal potentials for Na, K, and *h* channels were set as 55 mV, −90 mV, and − 30 mV, respectively. All calcium channels were modeled using the Goldman–Hodgkin–Katz (GHK) formulation (Goldman, 1943; Hodgkin & Katz, 1949), with default values of intracellular and extracellular calcium concentrations set as 50 nM and 2 mM, respectively. The evolution of cytosolic calcium concentration [Ca]_*c*_, defining its dependence on calcium current and its decay, was adopted from the formulation (Carnevale & Hines, 2006; Destexhe, Babloyantz, & Sejnowski, 1993; Narayanan & Johnston, 2010; Poirazi, Brannon, & Mel, 2003):

(1) $$\frac{d{\left[\mathrm{Ca}\right]}_{c}}{\mathrm{dt}}=-\frac{10000\;I_{\mathrm{Ca}}}{36\;\times \mathrm{dpt}\times F}+\frac{{\left[\mathrm{Ca}\right]}_{\infty }-{\left[\mathrm{Ca}\right]}_{c}}{\tau_{\mathrm{Ca}}}$$

where *F* represented Faraday's constant, *τ*_Ca_ = 160 ms defined the calcium decay constant in GCs (Eliot & Johnston, 1994), *dpt* = 0.1 μm was the depth of the shell into which calcium influx occurred, and [Ca]_*∞*_ = 50 nM is the steady‐state value of [Ca]_*c*_.

In performing MPMOSS on this GC base model, we used a search space spanning 38 active parameters associated with the nine active conductances and two parameters that defined the passive properties of the model (leak conductance, *g*_L_ = 1/*R*_m_ and *C*_m_). We generated 20,000 unique models by randomly picking the values of 40 parameters from independent uniform distributions that spanned the range for that specific parameter (Table 1). The multiple objectives of this MPMOSS strategy was with reference to bounds on nine different measurements computed for each of these 20,000 models, and the goal was to find models that had all nine measurements fall within their experimentally set bounds (Table 2). We found 126 models (~0.63% of the total population) to be valid in terms of achieving these multiple objectives, which were used as the heterogeneous GC population.

**Table 1:** Parameters and their ranges for granule cells

	Parameter	Symbol	Default	Testing range
h channel properties				
1	Maximal conductance (μS/cm^2^)	*h‐g*	5	2–12
2	Activation time constant of *I*_h_ (ms)	*h‐τ*_A_	39	30–50
3	*V*_1/2_ activation of *I*_h_ (mV)	*h‐V*_A_	–81	−70 to −90
*A*‐type K^+^ channel properties				
4	Maximal conductance (mS/cm^2^)	*KA‐g*	87	70–110
5	Activation time constant of KA (ms)	*KA‐τ*_A_	0.454	0.42–0.7
6	Inactivation time constant of KA (ms)	*KA‐τ*_I_	6.54	3–10
7	*V*_1/2_ activation of KA (mV)	*KA‐V*_A_	−55	−50 to −62
8	*V*_1/2_ inactivation of KA (mV)	*KA‐V*_I_	−73.1	−69 to −82
Delayed rectifier K^+^ channel properties				
9	Maximal conductance (μS/cm^2^)	*KDR‐g*	500	320–1,100
10	Activation time constant of KDR (ms)	*KDR‐τ*_A_	6.4	5–10
11	*V*_1/2_ activation of KDR (mV)	*KDR‐V*_A_	−44	−38 to −50
Fast Na^+^ channel properties				
12	Maximal conductance (mS/cm^2^)	*Na‐g*	18	16–50
13	Activation time constant of Na (μs)	*Na‐τ*_A_	50	42–56
14	Inactivation time constant of Na (ms)	*Na‐τ*_I_	3	2–6
15	*V*_1/2_ activation of Na (mV)	*Na‐V*_A_	−31	−30 to −40
16	*V*_1/2_ inactivation of Na (mV)	*Na‐V*_I_	−49	−43 to −55
Small conductance Ca^2+^‐dependent potassium (*SK*) channel properties				
17	Maximal conductance (mS/cm^2^)	*SK‐g*	5	1–12
18	*Ca*_1/2_ activation of SK (μM)	*SK‐C*_A_	4	1–8
19	Activation time constant of SK (ms)	*SK‐τ*_A_	214	195–250
20	Decay constant of calcium	*Ca‐τ*_decay_	160	95–206
Large conductance Ca^2+^‐activated potassium (*BK*) channel properties				
21	Maximal conductance (mS/cm^2^)	*BK‐g*	110	14–190
22	*C*_1/2_ activation of BK (μM)	*BK‐C*_A_	4	2–7
23	Activation time constant of BK (Ca^2+^‐dependent) (ms)	*BK‐Cτ*_A_	10	5–15
24	Activation time constant of BK (voltage‐dependent) (μs)	*BK‐τ*_A_	5	3–11
25	*V*_1/2_ activation of BK (mV)	*BK‐V*_A_	−28	−18 to −36
*L*‐type Ca^2+^ channel properties				
26	Maximal conductance (μS/cm^2^)	*CaL‐g*	700	105–800
27	Activation time constant of *L*‐type (μs)	*CaL‐τ*_A_	3	1–12
28	*V*_1/2_ activation of *L*‐type (mV)	*CaL‐V*_A_	−1.3	−5 to 7
*N*‐type Ca^2+^ channel properties				
29	Maximal conductance (μS/cm^2^)	*CaN‐g*	0.5	0.1–5
30	Activation time constant of *N* type (ms)	*CaN‐τ*_A_	0.6	0.1–1
31	Inactivation time constant of *N* type (ms)	*CaN‐τ*_I_	1,297	1,050–1,450
32	*V*_1/2_ activation of *N* type (mV)	*CaN‐V*_A_	−21	−30 to −10
33	*V*_1/2_ inactivation of *N* type (mV)	*CaN‐V*_I_	−40	−50 to −30
*T*‐type Ca^2+^ channel properties				
34	Maximal conductance (μS/cm^2^)	*CaT‐g*	0.7	0.5–10
35	Activation time constant of *T* type (ms)	*CaT‐τ*_A_	4	2–10
36	Inactivation time constant of *T* type (ms)	*CaT‐τ*_I_	7,665	6,800–8,400
37	*V*_1/2_ activation of *T* type (mV)	*CaT‐V*_A_	−36	−28 to −42
38	*V*_1/2_ inactivation of *T* type (mV)	*CaT‐V*_I_	−67	−75 to −58
Passive properties				
39	Specific membrane resistivity (kΩ cm^2^)	*R*_m_	38	30–42
40	Specific membrane capacitance (μF/cm^2^)	*C*_m_	1	0.8–1.2

**Table 2:** Experimental bounds for various granule cell measurements

	Measurement, unit	Symbol	Lower	Upper
1	Action potential amplitude, mV	*V*_AP_	95	115
2	Action potential threshold, mV	*V*_th_	−55	−40
3	Action potential half‐width, ms	*T*_APHW_	0.53	1.6
4	Fast after hyperpolarization, mV	*V*_fAHP_	−25	−3.4
5	Sag ratio	Sag ratio	0.9	1
6	Spike frequency adaptation	SFA	0.1	0.8
7	Input resistance, MΩ	*R*_in_	107	228
8	Firing frequency at 50 pA, Hz	*f*_50_	0	0
9	Firing frequency at 150 pA, Hz	*f*_150_	10	15

A similar MPMOSS strategy was used to generate a heterogeneous population of basket cells, whose geometry was set as a cylinder with 66 μm diameter and 66 μm length. The passive parameters of the BC base model were as follows: *V*_RMP_ = −65 mV, *R*_m_ = 7.1 kΩ cm^2^, *C*_m_ = 1 μF/cm^2^. Four different voltage‐gated ion channels (HCN, KA, NaF, and KDR) were introduced into the model, with the parameters set to match experimental measurements (Magee, 1998; Santhakumar et al., 2005). With these passive and active parametric values (Table 3), the *R*_in_ of the base BC model was 57 MΩ.

**Table 3:** Parameters and their ranges for basket cells

	Parameter	Symbol	Default value	Testing range
*h* channel properties				
1	Maximal conductance (μS/cm^2^)	*h‐g*	3	0.3–10
2	Activation time constant of *I*_h_ (ms)	*h‐τ*_A_	39	30–50
3	*V*_1/2_ activation of *I*_h_ (mV)	*h‐V*_A_	−81	−70 to −90
*A*‐type K^+^ channel properties				
4	Maximal conductance (mS/cm^2^)	*KA‐g*	0.4	0.1–1.5
5	Activation time constant of KA (ms)	*KA‐τ*_A_	11.549	5–15
6	Inactivation time constant of KA (ms)	*KA‐τ*_I_	11.69	10–15
7	*V*_1/2_ activation of KA (mV)	*KA‐V*_A_	−33	−28 to −38
8	*V*_1/2_ inactivation of KA (mV)	*KA‐V*_I_	−83	−80 to −90
Fast delayed rectifier K^+^ channel properties				
9	Maximal conductance (S/cm^2^)	*KDR‐g*	0.0017	0.0011–0.0025
10	Activation time constant of KDR (ms)	*KDR‐τ*_A_	2.16	1–4
11	*V*_1/2_ activation of KDR (mV)	*KDR‐V*_A_	−26.76	−20 to −30
Na^+^ channel properties				
12	Maximal conductance (mS/cm^2^)	*Na‐g*	200	90–300
13	Activation time constant of Na (ms)	*Na‐τ*_A_	0.066	0.055–0.075
14	Inactivation time constant of Na (ms)	*Na‐τ*_I_	3.99	2–8
15	*V*_1/2_ activation of Na (mV)	*Na‐V*_A_	−29	−20 to −35
16	*V*_1/2_ inactivation of Na (mV)	*Na‐V*_I_	−47.59	−40 to −55
Passive properties				
17	Specific membrane resistivity (Ω cm^2^)	*R*_m_	7,100	5,000–15,000
18	Specific membrane capacitance (μF/cm^2^)	*C*_m_	1	0.8–1.2

The stochastic search for BCs involved 16 parameters associated with the four voltage‐gated ion channels and two parameters defining passive membrane properties. Together, we picked 18 passive and active parametric values from independent uniform distributions (bounds are shown in Table 3), and generated 8,000 unique BC models. The physiological measurements that constituted the multiple objectives in defining the validity of BC models were the same as those for GCs, but with different experimentally derived ranges for each measurement (Table 4). This procedure yielded 54 valid BC models (~0.675% of the total population) with significant heterogeneity in each of the 18 intrinsic parameters that constructed them, and were used as the heterogeneous BC population. The experimental bounds on measurements for granule (Table 2) and basket (Table 4) cells were obtained from (Aradi & Holmes, 1999; Krueppel, Remy, & Beck, 2011; Lubke, Frotscher, & Spruston, 1998; Mott, Turner, Okazaki, & Lewis, 1997; Santhakumar et al., 2005).

**Table 4:** Experimental bounds for various basket cell measurements

	Measurement	Symbol	Lower	Upper
1	Action potential amplitude, mV	*V*_AP_	110	120
2	Action potential threshold, mV	*V*_th_	−51	−41
3	Action potential half‐width, ms	*T*_APHW_	0.53	1.5
4	Fast after hyperpolarization, mV	*V*_fAHP_	−27	−14
5	Sag ratio	Sag ratio	0.9	1
6	Spike frequency adaptation	SFA	0.9	1.04
7	Input resistance, MΩ	*R*_in_	45	65
8	Firing frequency at 50 pA, Hz	*f*_50_	0	0
9	Firing frequency at 150 pA, Hz	*f*_150_	30	50

### 2.2 Synaptic heterogeneity: Local network structure and randomization of connection strength

A network of 500 GCs and 75 BCs, with the GC:BC ratio constrained by experimental observations (Aimone et al., 2009), was constructed by randomly picking the valid models from the population of GCs and BCs obtained from MPMOSS. These 575 cells were distributed in a cylindrical neuropil of 156 μm diameter and 40 μm depth (Figure 2a), and matches the observed neuronal density (0.75 × 10^6^/mm^3^) in the DG region (Boss, Peterson, & Cowan, 1985). Although the default network size was 575 (Figure 2a), in testing scale invariance of our conclusions, in one set of simulations (Figure 12), we used a 115‐neuronal network made of 100 GCs and 15 BCs, again picked from their respective valid model populations. Irrespective of network size, local connectivity was set such that the probability of a BC to GC connection was 0.1, and that of a GC to BC connection was set as 0.05 (Aimone et al., 2009).

The GC → BC and BC → GC connections were modeled as synapses containing AMPA and GABA_A_ receptors, respectively. The GC → BC AMPA receptor current as a function of voltage (*v*) and time (*t*) was modeled, following the GHK convention (Goldman, 1943; Hodgkin & Katz, 1949; Narayanan & Johnston, 2010):

(2) $$I_{\text{AMPA}}\left(v,t\right)=I_{\text{AMPA}}^{\mathrm{Na}}\left(v,t\right)+I_{\text{AMPA}}^{K}\left(v,t\right)$$

where,

(3) $$I_{\text{AMPA}}^{\mathrm{Na}}\left(v,t\right)={\bar{P}}_{\text{AMPAR}}\;P_{\mathrm{Na}}\;s\left(t\right)\;\frac{{\mathrm{vF}}^{2}}{\mathrm{RT}}\;\left(\frac{{\left[\mathrm{Na}\right]}_{i}-{\left[\mathrm{Na}\right]}_{o}\exp\left(-\frac{\mathrm{vF}}{\mathrm{RT}}\right)}{1-\exp\left(-\frac{\mathrm{vF}}{\mathrm{RT}}\right)}\right)$$

(4) $$I_{\text{AMPA}}^{K}\left(v,t\right)={\bar{P}}_{\text{AMPAR}}\;P_{K}\;s\left(t\right)\;\frac{{\mathrm{vF}}^{2}}{\mathrm{RT}}\;\left(\frac{{\left[K\right]}_{i}-{\left[K\right]}_{o}\exp\left(-\frac{\mathrm{vF}}{\mathrm{RT}}\right)}{1-\exp\left(-\frac{\mathrm{vF}}{\mathrm{RT}}\right)}\right)$$

where *F* is the Faraday's constant, *R* is the gas constant, *T* is the temperature and ${\bar{P}}_{\text{AMPAR}}$ is the maximum permeability of AMPAR. *s*(*t*) governed the AMPAR kinetics and was set as follows:

(5) $$s\left(t\right)=a\;\left(\exp\left(-t/\tau_{d}\right)-\exp\left(-t/\tau_{r}\right)\right)$$

where *a* normalized *s*(*t*) such that 0 ≤ *s*(*t*) ≤ 1, *τ*_d_ (= 10 ms) represented the decay time constant, *τ*_r_ (= 2 ms) depicted the rise time, *P*_Na_ = *P*_K_, [Na]_*i*_ = 18 mM, [Na]_*o*_ = 140 mM, *K*_*i*_ = 140 mM, and *K*_*o*_ = 5 mM, leading to the AMPAR reversal potential to be ~0 mV. The BC → GC GABA_A_ receptor chloride current was modeled as (Mishra & Narayanan, 2015)

(6) $$I_{\text{GABAA}}^{\mathrm{Cl}}\left(v,t\right)={\bar{P}}_{\text{GABAAR}}\;s\left(t\right)\;\frac{{\mathrm{vF}}^{2}}{\mathrm{RT}}\left(\frac{{\left[\mathrm{Cl}\right]}_{i}-{\left[\mathrm{Cl}\right]}_{o}\exp\left(\mathrm{vF}/\mathrm{RT}\right)}{1-\exp\left(\mathrm{vF}/\mathrm{RT}\right)}\right)$$

where ${\bar{P}}_{\text{GABAAR}}$ was the maximum permeability of GABA_A_ receptor. *s*(t) was identical to that for AMPAR. [*Cl*]_*i*_ = 5 mM and [*Cl*]_*o*_ = 98 mM.

Simulations were performed for various combinations of synaptic permeability parameters ${\bar{P}}_{\text{AMPAR}}$ and ${\bar{P}}_{\text{GABAAR}}$. These parameters were maintained at a regime where the peak‐firing rate of GCs and BCs stayed within their experimental ranges of 4–10 Hz and 30–50 Hz, respectively (Leutgeb et al., 2007). We ensured that extreme parametric combinations where the cell ceased firing (because of depolarization‐induced block at one extreme or high inhibition at the other) were avoided. When homogeneous synaptic connectivity was used, all ${\bar{P}}_{\text{AMPAR}}$ and ${\bar{P}}_{\text{GABAAR}}$ were set to identical values across the network, with different sets of network simulations performed with different ${\bar{P}}_{\text{AMPAR}}$–${\bar{P}}_{\text{GABAAR}}$ combinations (Figure 7b). In introducing local synaptic heterogeneity, we picked ranges for ${\bar{P}}_{\text{AMPAR}}$ and ${\bar{P}}_{\text{GABAAR}}$ that satisfied the firing rate requirements above and picked values for ${\bar{P}}_{\text{AMPAR}}$ and ${\bar{P}}_{\text{GABAAR}}$ (for all synapses in the network) from independent uniform distributions spanning this range (Figure 7c). Such local synaptic heterogeneities could be consequent to baseline biological variability in presynaptic properties and postsynaptic receptor densities, differential dendritic processing of inputs owing to active and passive filtering, differential spine sizes consequent to the interaction between homo‐ and heterosynaptic spine plasticity and homeostatic regulation of overall synaptic drives (Aimone et al., 2014; Coulter & Carlson, 2007; Dieni et al., 2013; Dieni et al., 2016; Jedlicka, Benuskova, & Abraham, 2015; Jungenitz et al., 2018; Krueppel et al., 2011; Li et al., 2017; Mongiat, Esposito, Lombardi, & Schinder, 2009).

### 2.3 Neurogenesis‐induced structural heterogeneity in neuronal age

Populations of immature GCs and BCs (originating through adult neurogenesis) were obtained by subjecting the mature set of the corresponding valid models (obtained through MPMOSS) to structural plasticity. Specifically, the reduction in dendritic arborization and in the overall number of channels expressed in immature neurons (Aimone et al., 2014) was approximated by a reduction in the surface area (diameter) of the model neuron, using *R*_in_ as the measurement to match with experimental counterparts. Experimentally, *R*_in_ of immature cells has been measured to be in the range of 3–6 GΩ (Overstreet‐Wadiche, Bromberg, Bensen, & Westbrook, 2006; Pedroni, Minh do, Mallamaci, & Cherubini, 2014; Schmidt‐Hieber, Jonas, & Bischofberger, 2004). The impact of structural plasticity (through change in diameter) on neuronal excitability was assessed on the 126 valid GCs (Figure 8a) and 54 valid BCs (Figure 8a), and as expected (Johnston & Wu, 1995; Rall, 1977) *R*_in_ increased with reduction in diameter (Figure 8a). From these sensitivity analyses, we set the diameter for the immature GC and BC populations to be at 2–9 and 1–3 μm, respectively, to match the experimental *R*_in_ of 3–6 GΩ (Figure 8a). We set neuronal diameters to their default values (63 μm for GCs and 66 μm for BCs) in networks constructed only from mature cells. For networks constructed using only immature cells, the neuronal diameters were picked randomly from their respective immature ranges (GC: 2–9 μm; BC: 1–3 μm). We introduced an additional layer of neurogenesis‐induced structural heterogeneity in neuronal age, a scenario that is more physiologically relevant, by setting the diameters of GCs and BCs to random values picked from independent uniform distributions that spanned the respective immature‐to‐mature range of diameters (GC: 2–63 μm; BC: 1–66 μm).

### 2.4 Input‐driven afferent heterogeneities: External inputs from the entorhinal cortex

All neurons in the DG network constructed above received inputs from two different regions of entorhinal cortex (EC): one from medial entorhinal cortex (MEC) grid cells that transmitted spatial information and another from lateral entorhinal cortex (LEC), which provides contextual information (Anderson, Morris, Amaral, Bliss, & O'Keefe, 2007; Renno‐Costa, Lisman, & Verschure, 2010). Each neuron received *active* inputs from 5 different MEC cells and 5 different LEC cells, with inputs from MEC and LEC split at 50%–50%. In one set of simulations (Figure 11), these active inputs were scaled to 10 different MEC cells and 10 different LEC cells, with inputs from MEC and LEC split equally. In populations receiving homogeneous inputs, all 575 neurons in the DG network received *identical* inputs from the MEC and LEC. To account for the sparse and orthogonal connectivity from the EC to the DG, input‐driven afferent heterogeneities were incorporated by defining MEC and LEC inputs to be distinct for each GC and BC cell in the network. In this case, each GC and BC received independent sets of inputs from 5 MEC and 5 LEC cells. In other words, a set of 575 × (5 + 5) = 5,750 (total # neurons × [# MEC + # LEC]) distinct external inputs impinged on the network.

The current input from a single grid cell to DG cells was modeled as a hexagonal grid function defined as a sum of three two‐dimensional cosine functions (Solstad, Moser, & Einevoll, 2006):

(7) $$\psi \left(x,y\right)=\frac{2}{3}\left(\frac{\cos\left(g_{1}\right)+\cos\left(g_{2}\right)+\cos\left(g_{3}\right)}{3}+\frac{1}{2}\right)$$

where (*x*, *y*) represented the position of the virtual animal in the arena, and *g*_1_, *g*_2_, and *g*_3_ were defined as

(8) $$g_{1}=\frac{4\pi \lambda }{\sqrt{6}}[\left(\cos\left(\theta +\frac{\pi }{12}\right)+\sin\left(\theta +\frac{\pi }{12}\right)\right)\left(x-x_{0}\right)+\left(\cos\left(\theta +\frac{\pi }{12}\right)-\sin\left(\theta +\frac{\pi }{12}\right)\right)\left(y-y_{0}\right)]$$

(9) $$g_{2}=\frac{4\pi \lambda }{\sqrt{6}}[\left(\cos\left(\theta +\frac{5\pi }{12}\right)+\sin\left(\theta +\frac{5\pi }{12}\right)\right)\left(x-x_{0}\right)+\left(\cos\left(\theta +\frac{5\pi }{12}\right)-\sin\left(\theta +\frac{5\pi }{12}\right)\right)\left(y-y_{0}\right)]$$

(10) $$g_{3}=\frac{4\pi \lambda }{\sqrt{6}}[\left(\cos\left(\theta +\frac{3\pi }{4}\right)+\sin\left(\theta +\frac{3\pi }{4}\right)\right)\left(x-x_{0}\right)+\left(\cos\left(\theta +\frac{3\pi }{4}\right)-\sin\left(\theta +\frac{3\pi }{4}\right)\right)\left(y-y_{0}\right)]$$

where *λ* represents the grid frequency, *θ* represents the grid orientation, and *x*_0_, *y*_0_ were offsets in *x*, *y*, respectively. This hexagonal grid function was scaled to obtain the input from a single MEC cell (Figure 2h), with the scaling performed to set the relative contribution of MEC and LEC to the DG cells. MEC cell inputs were distinct in terms of the grid frequency (*λ*: 2–6 Hz) and grid orientation (*θ*: 0–360°), each sampled from respective uniform distributions.

For modeling LEC inputs to GCs and BCs (Renno‐Costa et al., 2010), we tiled the 1 m × 1 m arena into 25 squares (5 rows and 5 columns). For each LEC cell, a 5 × 5 matrix that was isomorphic to this tiled arena was generated with values randomly assigned from 0 to 1. Regions of the matrix with values in the range 0–0.5 were inactive, whereas active regions were those with values in the range 0.5–1. This matrix was convolved with a Gaussian kernel to smoothen the active–inactive transition segments (Renno‐Costa et al., 2010). Inputs from this LEC cell to the DG cell was then defined as the scaled value of this matrix corresponding to the (*x*, *y*) location on the arena, with the scaling tuned to set the relative contribution of MEC and LEC to the DG cells. Each LEC cell input was associated with a unique randomized matrix, representing different active and inactive regions (Figure 2h).

In one set of experiments (Figure 13), we tested the impact of introducing neurogenesis‐induced structural heterogeneity only in the GC population, leaving the BC population to be mature (range of diameters for GC was 2–63 μm and the diameter for all BC was set at 66 μm). There are several lines of evidence that the synaptic connectivity to immature neurons are low, and that this low connectivity counterbalances their high excitability (Dieni et al., 2013; Dieni et al., 2016; Li et al., 2017; Mongiat et al., 2009). To assess the impact of such reduced synaptic drive on response decorrelation, in one set of simulations (Figure 13), we reduced the overall afferent drive in scenarios that involved neurogenesis‐induced structural differences. This reduction was implemented by scaling the afferent drive in a manner that was reliant on the neuronal diameter, with lower diameter translating to larger reduction in the synaptic drive, and was adjusted toward the goal of reducing firing rate variability across the neuronal population. The effects of restricting neurogenesis‐induced structural heterogeneity to GC and of reducing synaptic drive to immature neurons were both assessed in simulations where afferent inputs were either identical or heterogeneous, and in the presence or absence of several other local heterogeneities (Figure 13).

### 2.5 Single neuron measurements

The subthreshold and suprathreshold responses of GCs were quantified based on nine measurements (Lubke et al., 1998): neuronal firing rate with a pulse current injection of 50 pA (*f*_50_) and 150 pA (*f*_150_), sag ratio, *R*_in_, action potential (AP) amplitude (*V*_AP_), AP threshold (*V*_th_), AP half‐width (*T*_APHW_), fast after hyperpolarization (*V*_fAHP_) and spike frequency adaptation (SFA). *R*_in_ was measured from the neuronal steady‐state voltage response to each of 11 different current pulses, injected with amplitudes ranging from −50 to 50 pA (for 1,000 ms) in steps of 10 pA (Figure 2c). The steady‐state voltage deflections from *V*_RMP_ were plotted as a function of the corresponding current injections to obtain a *V–I* plot. We fitted a straight‐line function to this *V–I* plot (Figure 2c), and the slope of this linear fit defined *R*_in_. Sag ratio was calculated as the ratio of the steady‐state voltage deflection to the peak voltage deflection recorded in response to a −50 pA (1,000 ms) current injection (Figure 2d).

All suprathreshold measurements were obtained from the voltage trace recorded in response to a 150 pA depolarizing current injection, with AP measurements obtained from the first spike of this trace. Firing frequency was calculated as number of spikes in response to 150 pA current injection for 1 s (Figure 2e). Spike frequency adaptation (SFA) was calculated as the ratio of the first interspike interval (ISI) to the last ISI (Figure 2e). The voltage in the AP trace corresponding to the time point at which the *dV*/*dt* crossed 20 V/s defined AP threshold (Figure 2f). AP half‐width was the temporal width measured at the half‐maximal points of the AP peak with reference to AP threshold (Figure 2f). AP amplitude was computed as the peak voltage of the spike relative to *V*_RMP_ (Figure 2g). Fast after hyperpolarization (*V*_fAHP_) was measured as the maximal repolarizing voltage deflection of the AP from threshold (Figure 2g).

### 2.6 Network analyses: Virtual animal traversal and assessment of channel decorrelation

A virtual animal was allowed to traverse a 1 m × 1 m arena, and the *x* and *y* coordinates of the animal's location translated to changes in the external inputs from the MEC and LEC. The direction (range: 0–360°) and distance per time step (velocity: 2.5–3.5 m/s) were randomly generated, and were updated every millisecond. The amount of time taken for the virtual animal to approximately cover the entire arena was around 1,000 s (Figure 2h). All simulations were performed for 1,000 s, with the spatiotemporal sequence of the traversal maintained across simulations to allow direct comparisons, with the initial position set at the center of the arena. After the network was constructed with different forms of heterogeneities and with the different local connection strength and external inputs, the spike timings of each GC and BC were recorded through the total traversal period of 1,000 s. Instantaneous firing rates for each of these cells were computed from binarized spike time sequences by convolving them with a Gaussian kernel with a default standard deviation (*σ*_FR_) of 2 s.

In the default network (500 GC and 75 BC cells), correlation matrices for the GCs (500 × 500) were constructed by computing Pearson's correlation coefficient of respective instantaneous firing rate arrays (each spanning 1,000 s). Specifically, the (*i*, *j*)th element of these matrices was assigned the Pearson's correlation coefficient computed between the instantaneous firing rate arrays of neuron *i* and neuron *j* in the network (to assess channel decorrelation; Figure 1a). As these correlation matrices are symmetric with all diagonal elements set to unity, we used only the lower triangular part of these matrices for analysis and representation. In assessing channel decorrelation, irrespective of the specific set of heterogeneities incorporated into the network, we first plotted the distribution of these correlation coefficients. In addition, we represented correlation coefficients from individual distributions as mean ± *SEM*, and used the Kolmogorov Smirnov test to assess significance of differences between distributions.

In assessing channel decorrelation as a function of input correlation, we first computed the total afferent current impinging on each neuron. As the total current was the same for scenarios where identical afferent inputs were presented, the input correlation across all neurons was set at unity. For the scenario where the afferent inputs were heterogeneous, pairwise Pearson correlation coefficients were computed for currents impinging on different DG neurons and were plotted against the corresponding response correlation (for the same pair). Output correlations in this plot were binned for different values of input correlation, and the statistics (mean ± *SEM*) of response correlation were plotted against their respective input correlation bins (Figure 10c). As the computed correlation coefficients between firing rate response of two distinct neurons was critically dependent on the value of *σ*_FR_ (Supporting Information, Figure S1), we computed response correlation for several different values of *σ*_FR_ to ensure that our conclusions were not artifacts of narrow parametric choices (Figure 13d–g).

### 2.7 Computational details

All simulations were performed using the NEURON simulation environment (Carnevale & Hines, 2006), at 34°C with an integration time step of 25 μs. Analysis was performed using custom‐built software written in Igor Pro programming environment (Wavemetrics). Statistical tests were performed in statistical computing language R (www.R-project.org).

## 3 RESULTS

In systematically delineating the impact of distinct forms of heterogeneities on channel decorrelation (Figure 1a), we constructed networks of 500 GCs and 75 BCs from respective conductance‐based model populations (Figure 2a,b). The heterogeneous conductance‐based model populations of GC and BC neurons were derived from independent stochastic search procedures that replicated 9 different electrophysiological measurements (Figure 2c–g) for each cell type (Tables 1, 2, 3, 4). These 575 cells were distributed in a cylindrical neuropil of 156 μm diameter and 40 μm depth (Figure 2a), with cell density and local connection probability between GCs and BCs (Figure 2b) matched with experimental equivalents. Each cell in the network received local circuit inputs from other BCs or GCs (Figure 2b) and external inputs (Figure 2h) from several cells in the medial (MEC) and lateral entorhinal cortices (LEC), which allowed it to fire (Figure 2i) at specific locations (Figure 2j) within the arena that the virtual animal traversed in randomized order (over the entire simulation period of 1,000 s).

### 3.1 Degeneracy in single neuron physiology of granule and basket cell model populations

We used a well‐established stochastic search strategy (Foster et al., 1993; Goldman et al., 2001; Prinz et al., 2004; Rathour & Narayanan, 2014) to arrive at populations of conductance‐based models for GCs and BCs. This exhaustive and unbiased parametric search procedure was performed on 40 parameters for GCs (Table 1), and 18 parameters for BCs (Table 3), involving ion channel properties derived from respective neuronal subtypes. Nine different measurements, defining excitability and action potential firing patterns (Figure 2 and Table 2), were obtained from each of the 20,000 stochastically generated unique GC models, and were matched with corresponding electrophysiological GC measurements. We found 126 of the 20,000 models (~0.63%) where all nine measurements were within these electrophysiological bounds (Table 2), and thus were declared as valid GC models. A similar procedure was used for BC cells, where 9 different measurements from 8,000 unique models were compared with corresponding electrophysiological BC measurements. Here, we found 54 of the 8,000 models (~0.675%) where all nine measurements were within electrophysiological bounds (Table 4), and declared them as valid BC models. The experimental bounds on physiological measurements for granule (Table 2) and basket (Table 4) cells were obtained from references (Aradi & Holmes, 1999; Krueppel et al., 2011; Lubke et al., 1998; Mott et al., 1997; Santhakumar et al., 2005).

**Figure 3:**
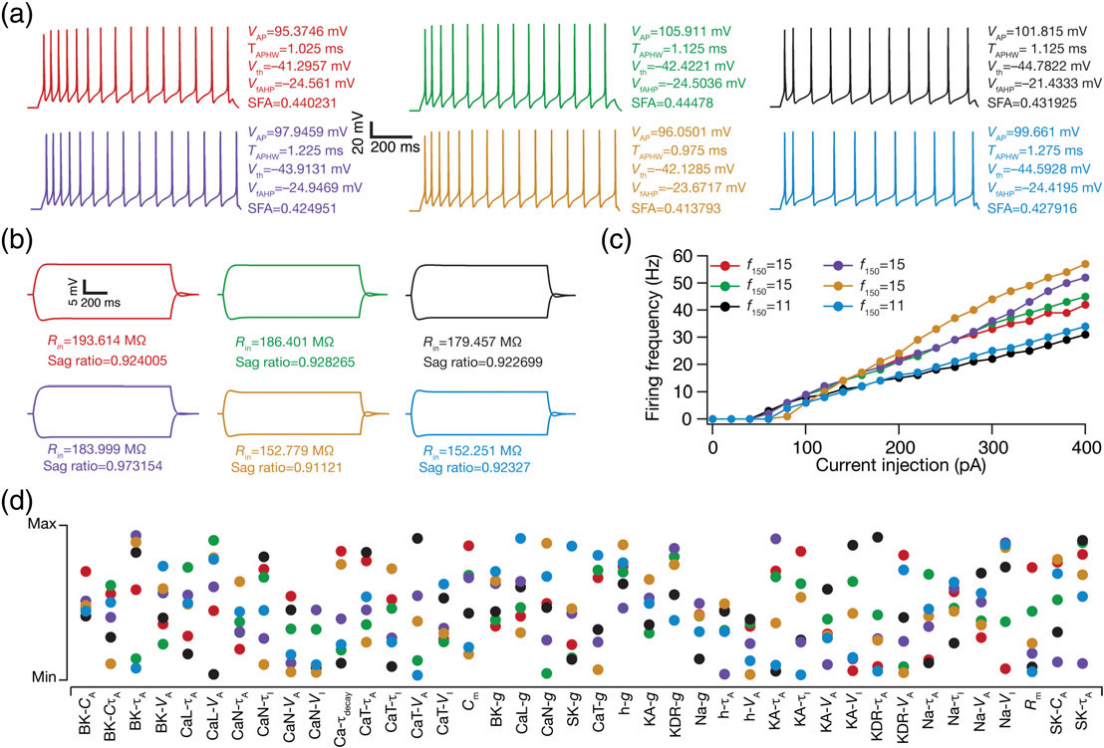
Illustration of cellular‐scale degeneracy in granule cell physiology with six randomly chosen valid models, where analogous functional characteristics were achieved through disparate parametric combinations. (a) Firing pattern of six randomly chosen valid GC models in response to 150 pA current injection with corresponding measurement values for action potential amplitude (*V*_AP_), action potential half‐width (*T*_APHW_), action potential threshold (*V*_th_), fast after hyperpolarization (*V*_fAHP_), and spike frequency adaptation (SFA). (b) Voltage traces of six valid GC models in response to −50 and 50 pA current injection, with associated measurement values for input resistance (*R*_in_) and sag ratio. Note that firing rate at 150 pA, *f*_50_, was zero for all models. (c) Firing frequency plots for six valid GC models in response to 0–400 pA current injections, indicating values of firing rate at 150 pA for each valid model. Note that all the 9 different measurements are very similar across these six models. (d) Distribution of the 40 underlying parameters in the six valid models, shown with reference to their respective min–max ranges. The color code of the dots is matched with the plots and traces for the corresponding valid models in a–c [Color figure can be viewed at wileyonlinelibrary.com]

**Figure 4:**
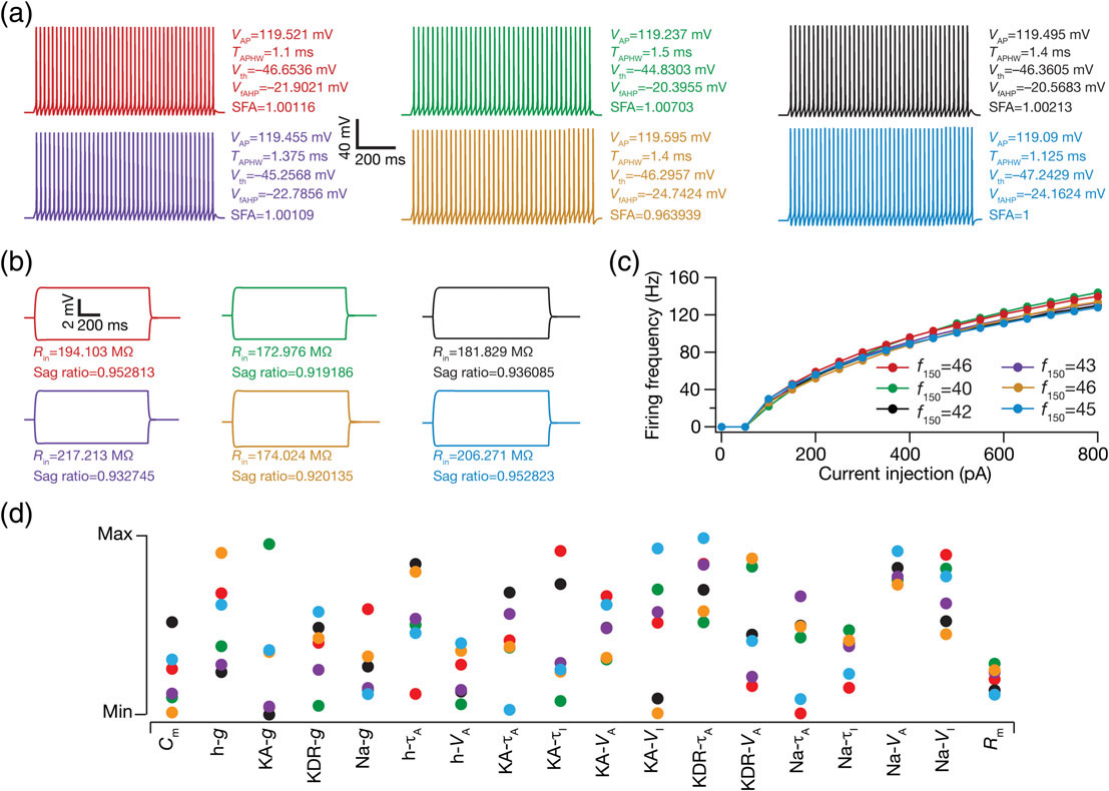
Illustration of cellular‐scale degeneracy in basket cell physiology with six randomly chosen valid models, where analogous functional characteristics were derived from disparate parametric combinations. (a) Firing pattern of six randomly chosen valid BC models in response to 150 pA current injection with corresponding measurement values for action potential amplitude (*V*_AP_), action potential half‐width (*T*_APHW_), action potential threshold (*V*_th_), fast after hyperpolarization (*V*_fAHP_), and spike frequency adaptation (SFA). (b) Voltage traces of six valid BC models in response to −50 and 50 pA current injection, with associated measurement values for input resistance (*R*_in_) and sag ratio. (c) Firing frequency plots for six valid BC models in response to 0–800 pA current injections, indicating values of firing rate at 150 pA for each valid model. (d) Distribution of underlying 18 parameters in the six valid BC models, shown with reference to their respective min–max ranges. The color code of the dot is matched with the plots and traces for the corresponding valid model in a–c [Color figure can be viewed at wileyonlinelibrary.com]

**Figure 5:**
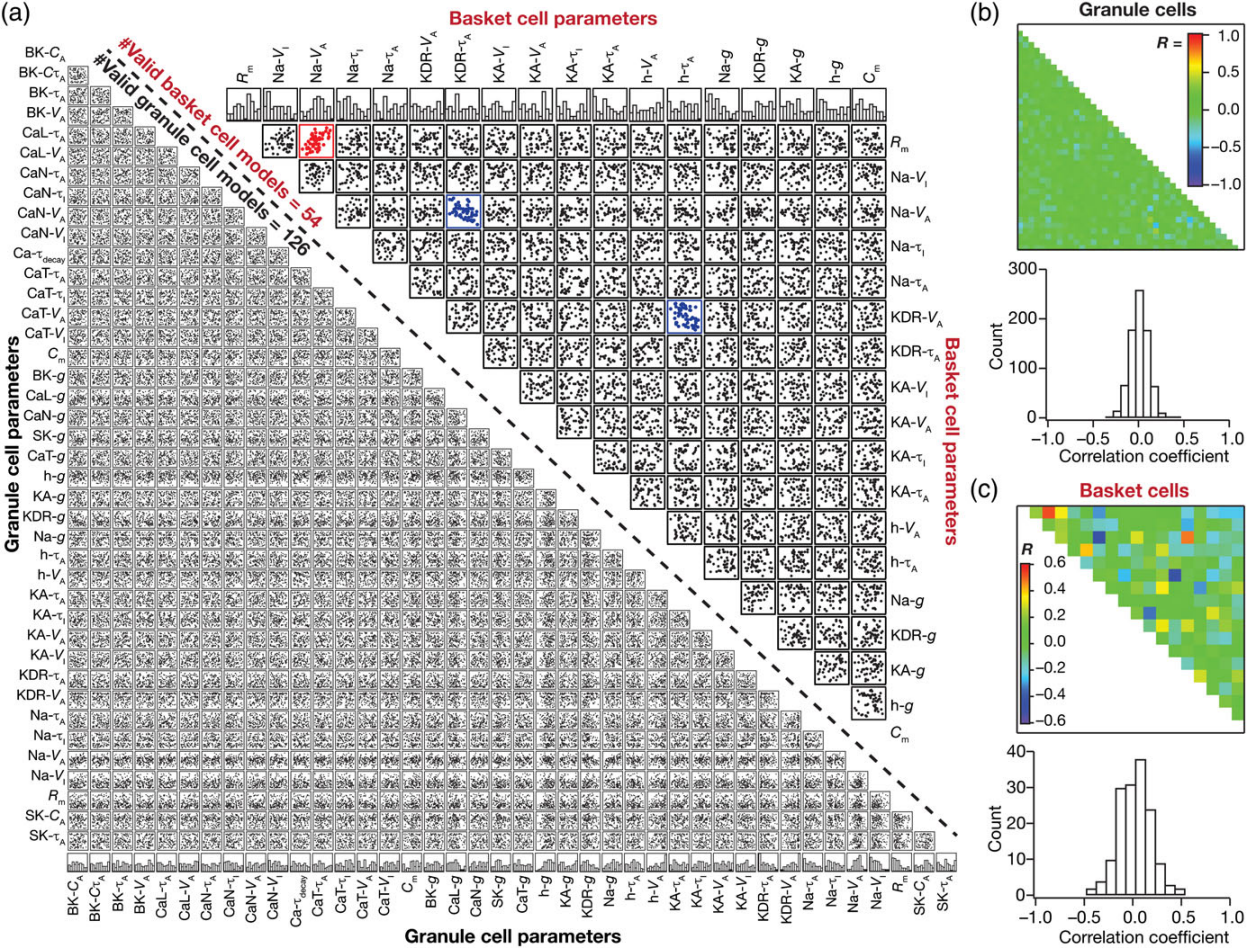
Independently heterogeneous populations of granule and basket cells exhibited cellular‐scale degeneracy with weak pair‐wise correlations of underlying parameters. (a) Left, lower triangular part of a matrix comprising pair‐wise scatter plots between 40 parameters underlying all valid GC models (*n* = 126). The bottom‐most row represents the histograms for corresponding parameters in the valid model population, showing all parameters spanning their respective min–max ranges. Right, upper triangular part of a matrix comprising pair‐wise scatter plots between 18 parameters underlying all valid BC models (*n* = 54). The topmost row represents the histograms for corresponding parameters in the valid model population, showing all parameters spanning their respective min–max ranges. The red scatter plots indicate that the value of correlation coefficient for the pair was >0.5, whereas the blue scatter plots denote pairs where the correlation coefficient value was <−0.5. (b) Top, heat map of correlation coefficient values for GC cells, corresponding to each scatter plot box depicted in a. Bottom, distribution of correlation coefficient values for the 780 unique pairs, of the 40 parameters, corresponding to scatter plots for GC parameters shown in a. (c) Same as (b) but for BC cells with 153 unique pairs of correlation coefficients (a) [Color figure can be viewed at wileyonlinelibrary.com]

**Figure 6:**
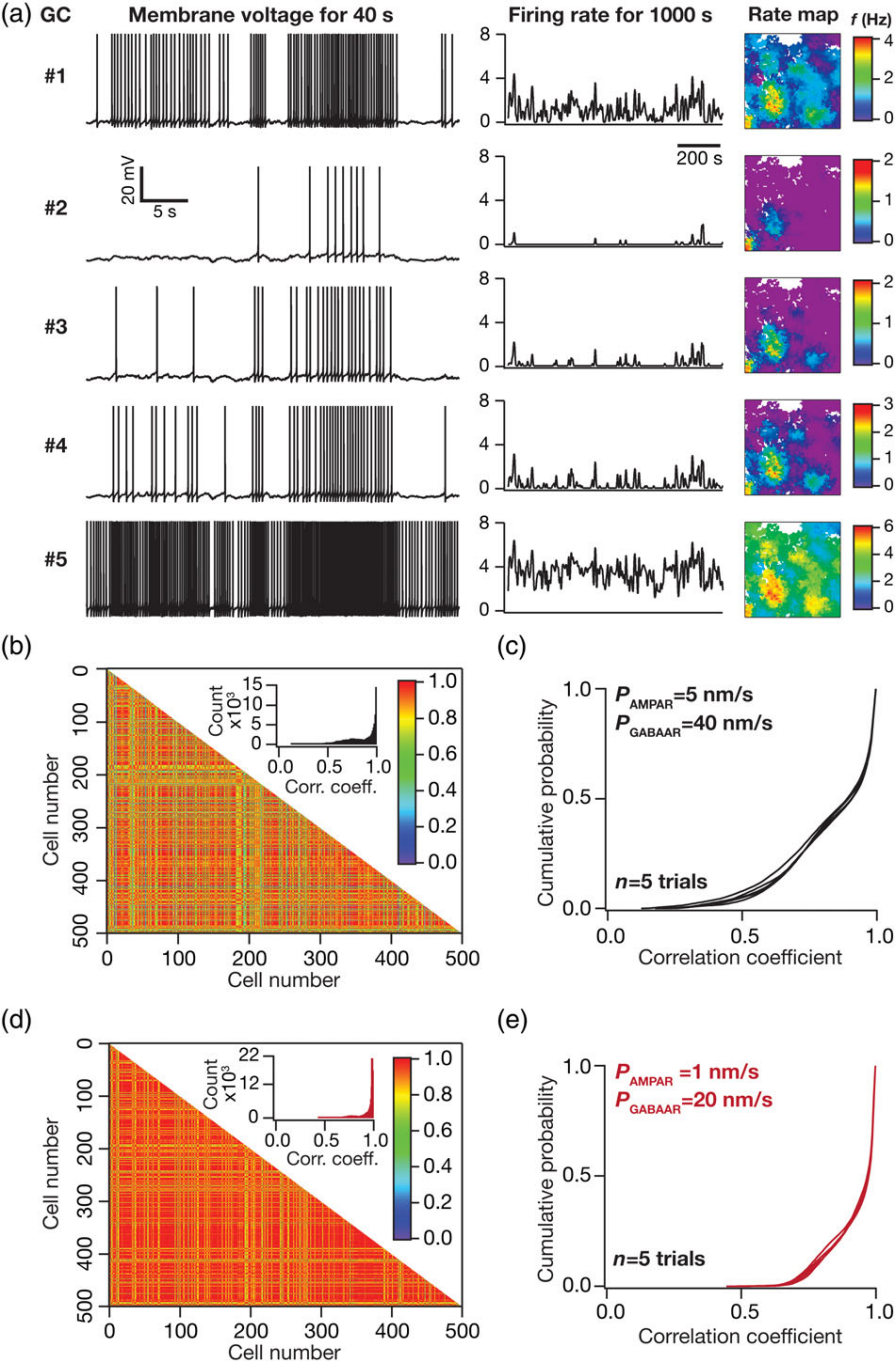
Heterogeneity in intrinsic neuronal excitability is a robust mechanism for achieving channel decorrelation through rate remapping of cellular responses. (a) Voltage traces (left), instantaneous firing rate (middle), and color‐coded rate maps (right; superimposed on the arena) for five different GCs in a network made of a heterogeneous GC and BC populations. (b) Lower triangular part of correlation matrix representing pair‐wise Pearson's correlation coefficient computed for firing rates of 500 GCs spanning the entire 1,000 s simulation period. Inset represents the histogram of these correlation coefficients. Note that there was no heterogeneity in the synaptic strengths of local connections, with *P*_AMPAR_ = 5 nm/s and *P*_GABAAR_ = 40 nm/s for all excitatory and inhibitory synapses, respectively. (c) Cumulative distribution of correlation coefficients represented in matrix in b. Plotted are distributions from five different trials of the simulation, with each trial different in terms of the cells picked to construct the network. (d,e) Same as (b,c), but with the synaptic strengths of local connections fixed at lower permeability values: *P*_AMPAR_ = 1 nm/s and *P*_GABAAR_ = 20 nm/s [Color figure can be viewed at wileyonlinelibrary.com]

Did the validation process place tight restrictions on model parameters that resulted in the collapse of all valid models to be near‐homogeneous equivalents with very little changes in their parametric values? To address this, we plotted model parameters of 6 GCs (Figure 3) and 6 BCs (Figure 4), which had near‐identical measurements values, and found the parametric values to spread through a wide span of the range used in the respective stochastic searches. To further validate this, we plotted histograms of each of the 40 GC model parameters and the 18 BC model parameters, and found them to spread through the entire span of their respective ranges (Figure 5a). These results demonstrated that the valid models were not near‐homogeneous parametric equivalents, but form heterogeneous populations of GCs and BCs that functionally matched their respective electrophysiological measurements, thereby unveiling cellular‐scale degeneracy in GC and BC neurons.

How did these neuronal populations achieve degeneracy? Did they achieve this by pair‐wise compensation across parameters, or was change in one parameter compensated by changes in several other parameters to achieve robust physiological equivalence? In answering this, we plotted pair‐wise scatter plots, independently on valid model parameters of the GC and BC populations (Figure 5a), and computed pair‐wise Pearson's correlation coefficients for each scatter plot (Figure 5b,c). We found that a vast majority of these pairs displayed very weak pair‐wise correlations (*R* ^2^ < 0.25; Figure 5b,c), suggesting that degeneracy in both populations was achieved through collective changes spanning several parameters.

### 3.2 Heterogeneities in neuronal intrinsic properties mediated decorrelation of neuronal responses to *identical* external inputs

Apart from demonstrating that robust cellular physiology could be achieved despite significant parametric variability, cellular‐scale degeneracy in these valid model populations provided an ideal manifestation of physiologically constrained intrinsic heterogeneities in the GC and BC model populations. Consequently, in defining the first layer of heterogeneity, we constructed a network of these heterogeneous populations with identical external inputs from the MEC and LEC and homogenous local synaptic connectivity.

We allowed the virtual animal to traverse the arena, recorded the voltage traces of all the GCs and BCs in this network, computed their firing rates and overlaid neuronal firing structure on the arena to observe the emergence of place fields (Figure 6a). To quantify the extent of decorrelation achieved through the introduction of intrinsic heterogeneities, we computed instantaneous firing rates of all neurons in the network across the entire traversal period (Figure 6a) and calculated pair‐wise Pearson's correlation coefficients across these firing rate arrays for all neurons (Figure 6b). If the network were composed of a homogeneous population of GCs and BCs receiving *identical* inputs, then the responses of all GCs would be identical to each other, with all pair‐wise correlation coefficients set at unity. However, owing to heterogeneous intrinsic excitability of individual neurons, their responses exhibited significant differences, especially in terms of overall firing rate at individual place fields (Figure 6a), even with identical external inputs and homogeneous local synaptic weights. Such dissimilarity in neuronal firing rate response emerges from two distinct manifestations of intrinsic heterogeneity. First, certain periods of identical synaptic inputs would be subthreshold for neurons with lower excitability (e.g.*,* Cell #2 in Figure 6a), but would be suprathreshold for neurons with relatively higher excitability (e.g., Cell #5 in Figure 6a), thereby manifesting as changes in firing rate or in the emergence of place fields at specific locations (Lee, Lin, & Lee, 2012). These observations suggest that DG neurons could undergo rate remapping (Leutgeb et al., 2007; Renno‐Costa et al., 2010) merely as a consequence of plasticity in intrinsic excitability. Second, although the numbers and synaptic weights of excitatory or inhibitory synapses received by neurons were identical, the patterns of activation of these synapses would be different across neurons as a consequence of significant variability in their respective presynaptic neuronal firing (Figure 6a).

Consequent to such variability in firing responses of this intrinsically heterogeneous population of neurons, we found the distribution of correlation coefficients of instantaneous firing rates to be significantly (Kolmogorov–Smirnov, KS test; *p* < .001) different from an all‐unity distribution representative of identical responses achieved in the absence of intrinsic variability (Figure 6b,c). Next, we repeated these simulations with different combinations of excitatory and inhibitory synaptic weights, setting all local synapses to the same value, and computed cumulative histograms of firing rate correlation coefficients (Figure 6d,e). We found a significant shift (Figure 6a vs e; KS test; *p* < .001) in the level of decorrelation with different combinations of synaptic weights.

### 3.3 Synaptic heterogeneity modulates decorrelation of neuronal responses to *identical* external inputs

**Figure 7:**
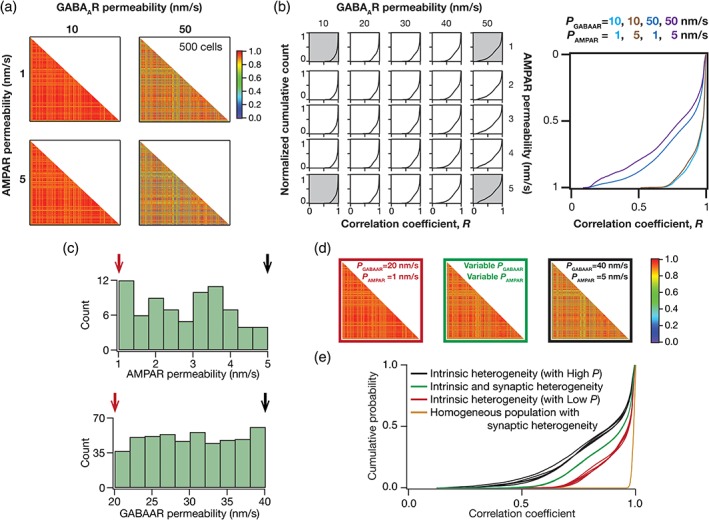
Heterogeneities in the strength of local network connections modulate channel decorrelation, with increase in inhibitory synaptic strength enhancing network decorrelation. (a) Lower triangular part of correlation matrix representing pair‐wise Pearson's correlation coefficient computed for firing rates of 500 GCs. Note that there was no heterogeneity in the synaptic strengths of local connections, with AMPAR and GABAAR permeability across local network synapses set at fixed values. Shown are four different correlation matrices, with *P*_AMPAR_ (1 or 5 nm/s) and *P*_GABAAR_ (10 or 50 nm/s) fixed at one of the two values. (b) Left, cumulative distribution of correlation coefficients for firing rates of 500 GCs, computed when the simulations were performed with different sets of fixed values of *P*_AMPAR_ (spanning 1–5 nm/s) and *P*_GABAAR_ (spanning 10–50 nm/s). The gray‐shaded plots on the extremes were computed from corresponding matrices shown in (a). Right, cumulative distributions of correlation coefficients corresponding to the gray‐shaded plots on the left, to emphasize the impact of synaptic heterogeneity on decorrelation. (c) Distribution of *P*_AMPAR_ and *P*_GABAAR_ in a network of heterogeneous GC and BC populations, constructed with heterogeneity in local synaptic strengths as well. Each AMPA and GABA_A_ receptor permeability was picked from a uniform distribution that spanned the respective ranges. The color codes of arrows and plots correspond to cases plotted in (d,e). (d) Lower triangular part of correlation matrices representing pair‐wise Pearson's correlation coefficient computed for firing rates of 500 GCs. For the right and left matrices, which are the same plots as in Figure 6c,e, respectively, there was no synaptic heterogeneity, with *P*_AMPAR_ and *P*_GABAAR_ set at specified fixed values for all excitatory and inhibitory synapses. The matrix represented in the center was computed from a network endowed with intrinsic and synaptic heterogeneity (shown in c). (e) Cumulative distribution of correlation coefficients represented in matrices in (d). Plotted are distributions from five different trials of each configuration. Note that except for the homogenous population, all three configurations were endowed with intrinsic heterogeneity. The configurations “intrinsic + synaptic heterogeneity” and “homogeneous + synaptic heterogeneity” had randomized synaptic permeabilities; for the other two configurations, the synaptic strengths were fixed at specific values: high *P*, *P*_AMPAR_ = 5 nm/s, and *P*_GABAAR_ = 40 nm/s; low *P*, *P*_AMPAR_ = 1 nm/s, and *P*_GABAAR_ = 20 nm/s [Color figure can be viewed at wileyonlinelibrary.com]

Motivated by observations on the role of the local synaptic weights in modulating response decorrelation, we systematically assessed the impact of altering the excitatory and inhibitory synaptic weights on the correlation histograms. As a first step, the network was endowed with intrinsic heterogeneities and all local synaptic weights were identical but were assigned different values across different simulations (Figure 7a,b). Although increases in either excitatory or inhibitory weights significantly enhanced the level of response decorrelation, the impact of increasing inhibitory weights had a dominant impact on decorrelating network responses (Figure 7a,b) emphasizing the critical role of local inhibitory neurons in defining response decorrelation in excitatory neurons (Aimone et al., 2014; Coulter & Carlson, 2007; Dieni et al., 2013).

Would introduction of synaptic heterogeneities, where different synapses in the local network assume distinct values, further enhance neuronal response decorrelation? To test this, we assigned weights of excitatory and inhibitory synapses in the local network to randomized values picked from respective uniform distributions (Figure 7c–e). Surprisingly, we found that introduction of synaptic heterogeneity did not enhance the level of response decorrelation, but allowed response decorrelation to express at a level that was within the bounds set by extreme values of identical synaptic weights (Figure 7e). Importantly, the level of decorrelation achieved by the introduction of local synaptic heterogeneity into a homogeneous population (no intrinsic heterogeneity) of GCs and BCs was significantly minimal compared to that achieved by the mere presence of intrinsic heterogeneity (Figure 7e). Together, although the introduction of synaptic heterogeneity critically modulated the level of response decorrelation, these results suggest intrinsic heterogeneity as the dominant form among intrinsic and synaptic forms of heterogeneities in mediating channel decorrelation.

### 3.4 Adult neurogenesis‐induced structural heterogeneity in neuronal age enhances decorrelation of neuronal responses to *identical* external inputs

A prominent hypothesis on the specific functions of adult neurogenesis in DG neurons is on their role in response decorrelation. One part of the rationale behind this hypothesis is the distinct excitability properties of new neurons that provide an additional layer of heterogeneity (Aimone et al., 2010; Aimone et al., 2011; Aimone et al., 2014; Deng et al., 2010; Kropff et al., 2015; Schmidt‐Hieber et al., 2004; Wang et al., 2000; Yassa & Stark, 2011). Although there are lines of evidence linking adult neurogenesis to response decorrelation, the specific role of new neurons and the additional layer of heterogeneity introduced by them in regulating channel decorrelation has not been systematically assessed.

**Figure 8:**
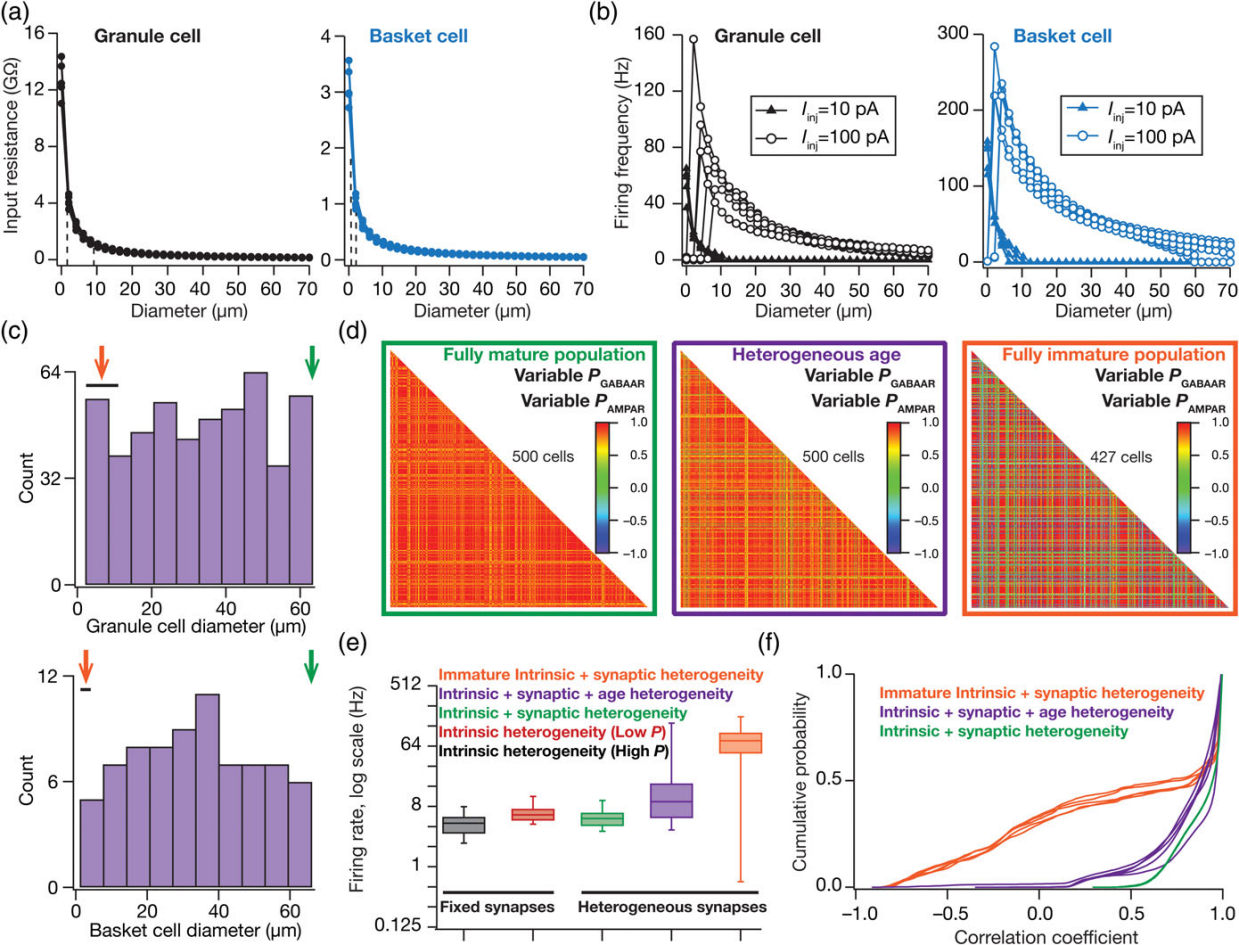
Incorporation of neurogenesis‐induced structural heterogeneity in neuronal age enhances channel decorrelation in a network of neurons receiving *identical* inputs. (a) Input resistance of the 126 GCs (left) and 54 BCs (right) plotted as a function of diameter of cell. Dotted lines represent the range for immature cell diameters (2–9 μm for GC and 1–3 μm for BC), obtained from ranges of experimentally obtained input resistance values in immature cells. (b) Firing frequency plotted as a function of diameter in response to 10 pA (closed triangles) and 100 pA (open circles) current injections into the 126 GCs (left) and 54 BCs (right). (c) Distribution of GC (top) and BC (bottom) diameters in a network of heterogeneous GC and BC populations, constructed with heterogeneity in local synaptic strengths and in the age of the neurons. The diameter of each GC and BC in the network was picked from a uniform distribution that spanned respective ranges. The color codes of arrows and plots correspond to fully mature network (green; large diameters), fully immature network (orange; small diameters), and mixed network (purple; variable diameters) cases plotted in (d–f). (d) Lower triangular part of correlation matrices representing pair‐wise Pearson's correlation coefficient computed for firing rates of all GCs. The matrix corresponding to the fully mature population is the same as that in Figure 7d, with the same color code. Note that all three networks were endowed with intrinsic and synaptic heterogeneity, with changes only in the neuronal age. (e) Firing rates, represented as quartiles, of all GCs plotted for the different networks they resided in. (f) Cumulative distribution of correlation coefficients represented in matrices in (d). Plotted are distributions from five different trials of each configuration [Color figure can be viewed at wileyonlinelibrary.com]

To introduce neurogenesis‐induced heterogeneity into our network, we noted that the excitability of new born neurons in the DG, which could mature to either GCs or BCs, is higher as a consequence of lower surface area reflective of the diminished arborization of immature neurons (Aimone et al., 2014; Liu et al., 2003; Schmidt‐Hieber et al., 2004; Wang et al., 2000). To quantitatively match the excitability properties of these neurons, we introduced structural plasticity by reducing the surface area of the valid GC and BC models (Figure 8) through reduction of their diameter. This reduction in surface area expresses as an increase in input resistance (Esposito et al., 2005; Rall, 1977; Schmidt‐Hieber et al., 2004) in each of these neurons (Figure 8a), which in turn translates to increase in firing rate (Figure 8b).

With the ability to introduce intrinsic, synaptic, and neurogenesis‐induced forms of heterogeneity into our network, we analyzed three distinct networks (fully mature, fully immature, and variable age) to specifically understand the role of neurogenesis‐induced heterogeneity on channel decorrelation to identical inputs. All three networks were endowed with intrinsic and synaptic heterogeneities receiving afferent inputs from the same arena (Figures 6 and 7), and the distinction between the three cases was only with reference to neuronal age (Figure 8d). In comparing the firing rates of the GCs for different network configurations, we found that the firing rates of all GCs were comparable for all cases where neurogenesis‐induced heterogeneities were absent. However, with the introduction of neurogenesis, especially in the scenario where all cells were immature, the firing rates increased and spanned a larger range. In the more physiologically relevant scenario of heterogeneous cellular age, although the firing rates spanned a larger range, a significant proportion of them were in the low firing regime characteristic of GCs (Figure 8e).

We found that the level of channel decorrelation in the fully immature network was significantly (KS test; *p* < .001) higher than that achieved in the fully mature network (Figure 8f). This is to be expected because the structural heterogeneity (effectuated by changes in diameter) would amplify the inherent intrinsic heterogeneity of neurons in the network, thereby enhancing the beneficiary effects of intrinsic heterogeneity that we had observed earlier (Figure 6). Importantly, reminiscent of our results with the introduction of synaptic heterogeneity (Figure 7), in the network that was endowed with variability in neuronal age, the level of decorrelation was intermediate between that obtained with the fully mature and the fully immature networks (Figure 8f). Together, these results demonstrate that neurogenesis‐induced variability in neuronal response properties adds an additional layer of structural heterogeneity in the DG network, and enhances channel decorrelation to *identical* external inputs. These results also demonstrate that among the three local heterogeneities assessed thus far, neurogenesis‐induced structural heterogeneities form the dominant heterogeneity, capable of inducing a much larger response decorrelation (compared to the input correlation set at 1, consequent to the identical nature of afferent inputs) compared to either synaptic or baseline intrinsic heterogeneities. Together, our experimental design involving systematic incorporation of biophysical, synaptic, and structural heterogeneities allowed us to specifically demonstrate a hierarchy of heterogeneities—synaptic, intrinsic, and neurogenesis‐induced structural, in increasing order of dominance when they coexpress—in effectuating channel decorrelation.

### 3.5 Input‐driven heterogeneity mediated by sparseness of afferent connectivity is a dominant regulator of channel decorrelation

An important defining characteristic of the rodent DG network is the sparseness of the afferent connectivity matrix that is reflective of massive convergence and divergence reflecting the small (~94,000) number of layer II EC cells (Gatome, Slomianka, Lipp, & Amrein, 2010; Mulders, West, & Slomianka, 1997) that project to a large (~1.2 million) number of DG cells (Rapp & Gallagher, 1996; West, Slomianka, & Gundersen, 1991), resulting in unique, sparse, and orthogonal set of afferent external inputs impinging on each GC (Aimone & Gage, 2011; Anderson et al., 2007; Li et al., 2017). Thus far in our analysis, in an effort to delineate the impact of three distinct forms of heterogeneity, we used an artificial construct where all neurons in the network received identical inputs. To assess the impact of this fourth form of afferent input‐driven heterogeneity, we introduced divergence in the set of EC neurons that project onto each GC and BC. This implied that each GC and BC now received distinct sets of EC inputs.

**Figure 9:**
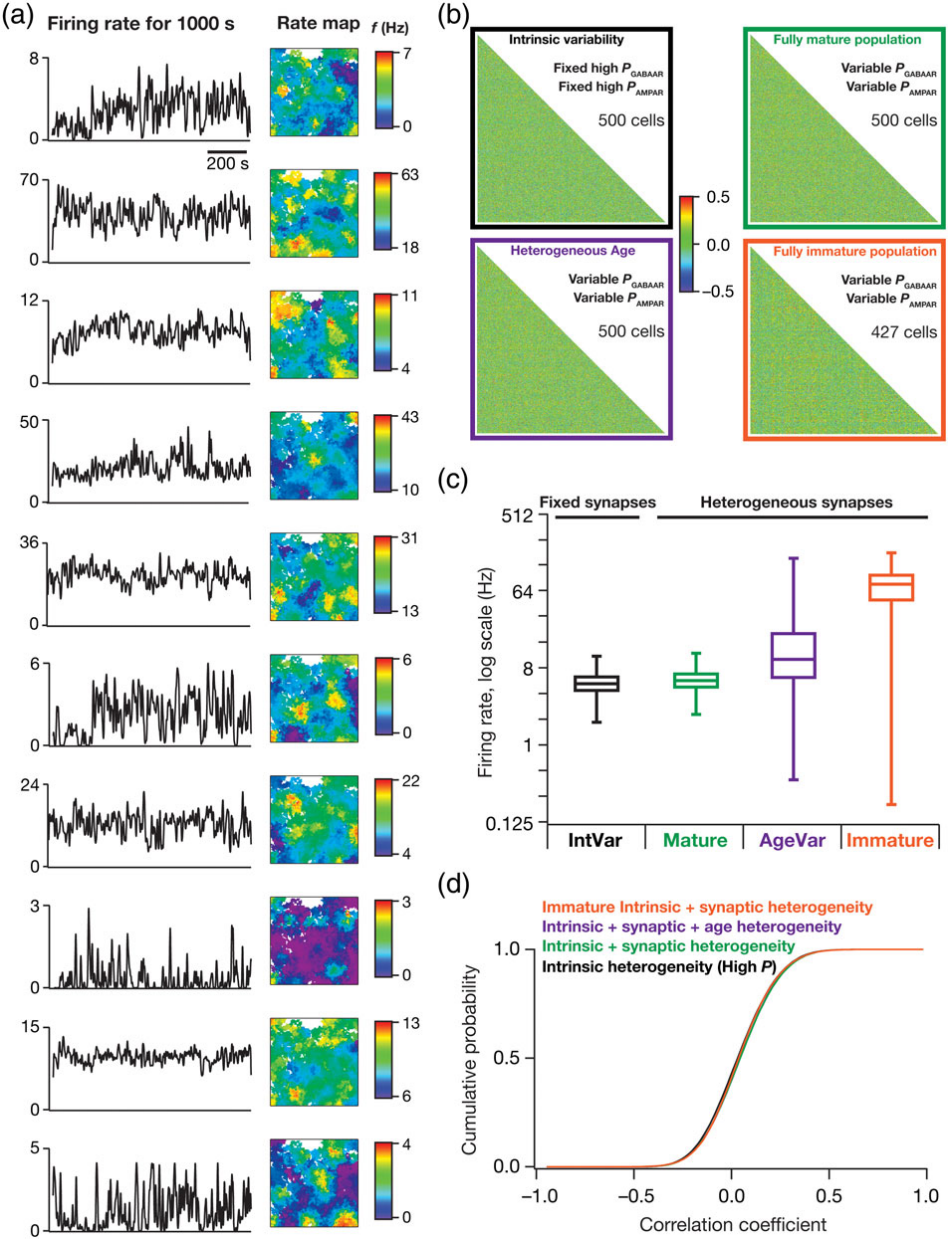
Heterogeneous external connectivity is the dominant form of variability that drives channel decorrelation in a network endowed with intrinsic, synaptic, and age heterogeneities. (a) Instantaneous firing rate (left) and color‐coded rate maps (right; superimposed on the arena) for 10 different GCs in a network endowed with intrinsic, synaptic, age, and input‐driven forms of heterogeneities. (b) Lower triangular part of correlation matrices representing pair‐wise Pearson's correlation coefficient computed for firing rates of all GCs. The four different matrices correspond to networks endowed with different sets of heterogeneities. (c) Firing rates, represented as quartiles, of all the GCs plotted for the different networks they resided in. Color codes for the specific set of heterogeneities included into the network are the same as those in Panel b above. (d) Cumulative distribution of correlation coefficients represented in matrices in (b) [Color figure can be viewed at wileyonlinelibrary.com]

**Figure 10:**
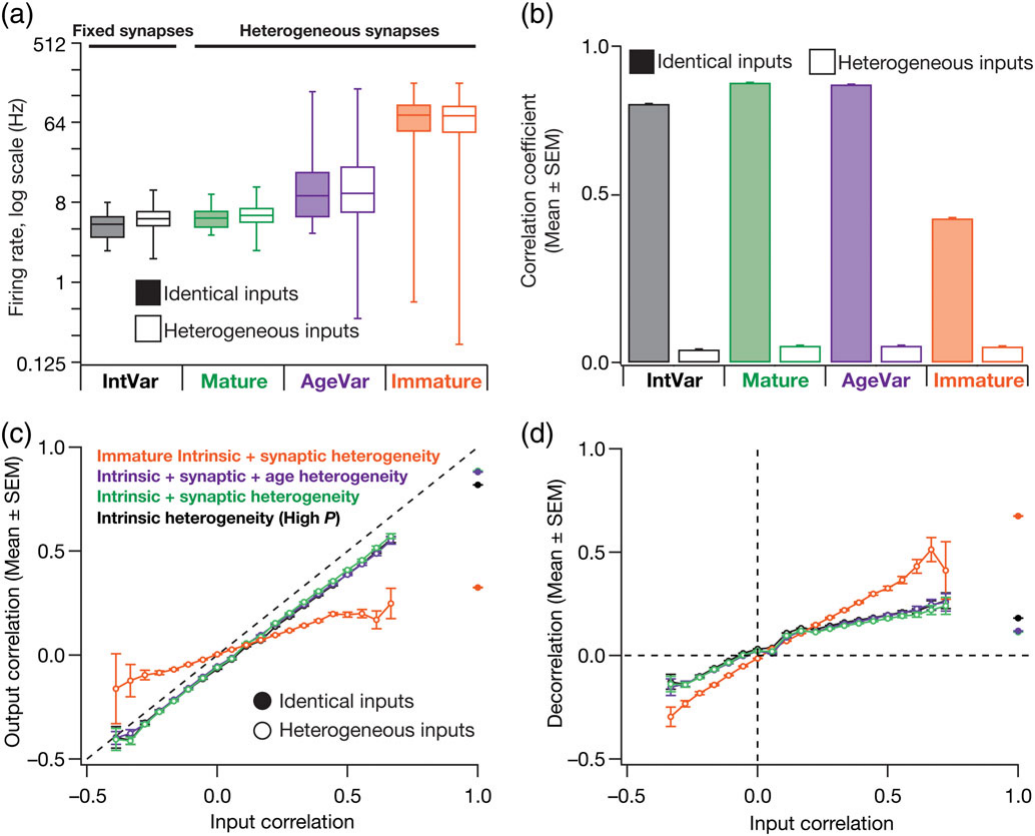
Afferent heterogeneities dominate channel decorrelation when they are coexpressed with other local‐network heterogeneities. (a) Firing rates, represented as quartiles, of all the GCs plotted for the different networks (heterogeneous vs identical input) they resided in. Color codes for the specific set of heterogeneities incorporated into the network are the same as those in Figure 9b. (b) Statistical (mean ± *SEM*) comparison of correlation coefficients obtained with networks, endowed with distinct forms of heterogeneities, receiving identical (solid boxes; derived from Figure 8f) versus variable (open boxes; derived from Figure 9d) external inputs. (c) Response (output) correlation plotted as a function of input correlation. Output correlations are the same as those plotted in Figure 8f (identical inputs) and Figure 9d (heterogeneous inputs). The corresponding input correlations represented Pearson's correlation coefficients computed for afferent current inputs onto individual neurons as the virtual animal traversed the arena. Note that the input correlation for identical input case is 1 with mean output correlation plotted correspondingly for identical case. (d) The difference between input correlation and respective output correlation (for individual pairs of neurons) plotted as “decorrelation” for the data represented in (c) [Color figure can be viewed at wileyonlinelibrary.com]

As a consequence of distinct set of inputs impinging on each GC, the firing fields were distinct across different GCs (Figure 9a), unlike the near‐identical firing fields (except for differences in firing frequency or threshold for firing) in the case where neurons received identical inputs (Figure 6a). Importantly, when we analyzed pair‐wise correlation of firing rates across different neurons, we found that the correlation coefficients were lower irrespective of the presence or absence of different forms of heterogeneity (Figures 9b and 10b). The overall firing rate distributions obtained with either identical (Figure 8e) or heterogeneous (Figure 9c) afferent inputs were similar (Figure 10a), thereby ruling out changes in firing rate as a possible cause for the observed differences in correlation coefficients.

When we plotted the cumulative distributions of correlation coefficients obtained with the introduction of distinct forms of local network heterogeneities, we found them to significantly overlap with each other (Figure 9d). This is in stark contrast to the network receiving identical external inputs (Figures 7e and 8f), where introduction of each of intrinsic, synaptic, and neurogenesis‐induced heterogeneities enhanced or altered the level of response decorrelation, with a well‐defined hierarchy among these heterogeneities (Figures 6, 7, 8). The negligible impact of the intrinsic or synaptic or age heterogeneities on the overall level of response decorrelation achieved in the presence of input‐driven heterogeneities unveiled the dominance of heterogeneities driven by afferent connectivity in determining response decorrelation (Figure 9d). To directly analyze channel decorrelation in networks endowed with distinct forms of heterogeneities, we plotted output response correlation against the respective input correlation values. Shown are output decorrelation values obtained for identical inputs (where input correlation values would be uniformly unity; Figure 10c, solid circles), demonstrating the differential impact of local network heterogeneities on channel decorrelation (values of correlation coefficients from Figure 8f). When output correlations were binned across different values of input correlation coefficients for the case where heterogeneous afferent inputs were presented to the network, we found the amount of channel decorrelation achieved in the presence of distinct local heterogeneities to be similar (Figure 10c,d, open circles). Specifically, the amount of decorrelation achieved in a network endowed with additional layers of synaptic and neurogenesis‐induced structural variability was not distinct from that achieved with a network endowed only with intrinsic heterogeneities (Figure 10c,d, open circles). Although the overall distributions of correlation coefficients for the fully immature population (an artificial construct) seemed similar when they received heterogeneous inputs (Figure 9d), the corresponding input–output correlation plots showed significantly higher decorrelation (Figure 10c,d, open circles), an observation that would be addressed later.

### 3.6 Sensitivity analyses confirmed the dominance of afferent heterogeneities in the emergence of channel decorrelation

**Figure 11:**
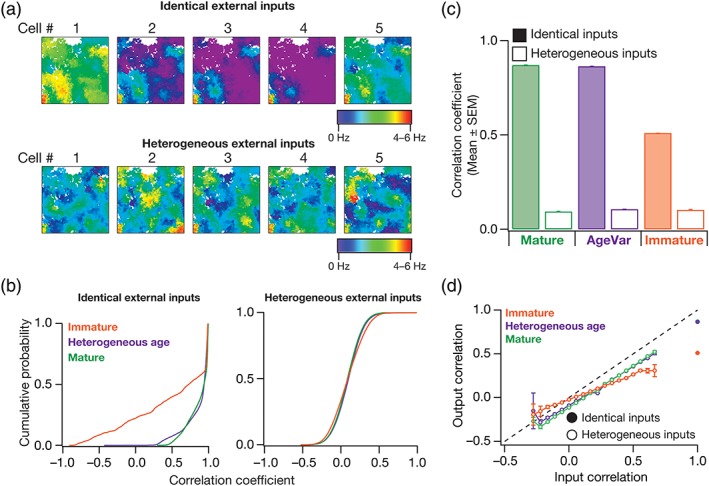
Heterogeneous afferent connectivity remains the dominant form of heterogeneity towards achieving channel decorrelation despite increase in the number of afferent inputs from EC. (a) Firing rate maps of five different GCs in a network made of a heterogeneous population of 500 GCs and 75 BCs, shown for cases when the network's external inputs were identical (top row) and heterogeneous (bottom row). (b) Cumulative distribution of response correlation coefficients represented for identical (left) and heterogeneous (right) external inputs. (c) Statistical (mean ± *SEM*) comparison of correlation coefficients obtained with networks endowed with distinct forms of heterogeneities, receiving identical (solid boxes; derived from panel b, left) versus heterogeneous (open boxes; derived from panel b, right) external inputs. (d) Response (output) correlation plotted as a function of input correlation for identical and heterogeneous external inputs [Color figure can be viewed at wileyonlinelibrary.com]

**Figure 12:**
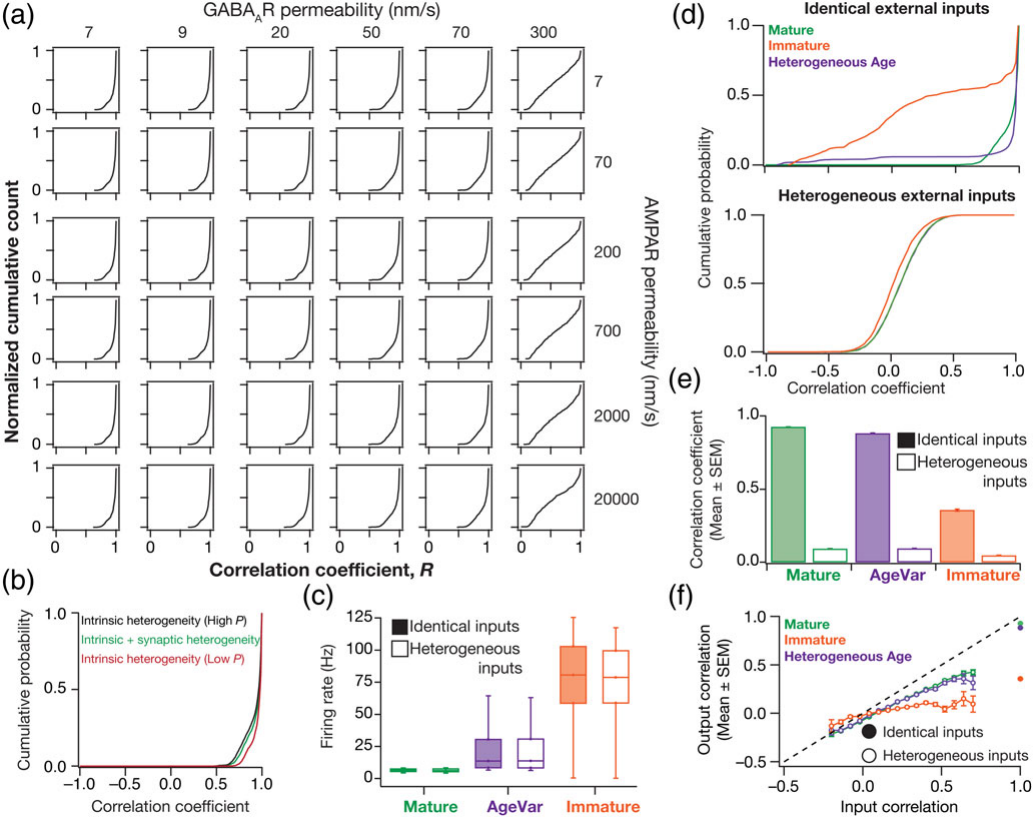
Heterogeneous afferent connectivity remains the dominant form of heterogeneity toward achieving channel decorrelation in a small DG network. (a) Cumulative distribution of correlation coefficients for firing rates of 100 granule cells, computed when the simulations were performed with different sets of fixed values of *P*_AMPAR_ (spanning 0.007–20 μm/s) and *P*_GABAAR_ (spanning 7–300 nm/s). These simulations were performed in networks constructed with heterogeneous populations of 100 GCs and 15 BCs, with fixed synaptic strengths. (b) Cumulative distribution of pair‐wise correlation coefficients computed from granule cell firing rates in networks constructed with different forms of heterogeneities. Note that all three configurations were endowed with intrinsic heterogeneities (heterogeneous GC and BC populations), and all cells in the network received identical external inputs. The “intrinsic + synaptic heterogeneity” configuration had randomized synaptic permeabilities; for the other two configurations, the synaptic strengths were fixed at specific values: high *P*, *P*_AMPAR_ = 700 nm/s, and *P*_GABAAR_ = 70 nm/s; low *P*, *P*_AMPAR_ = 7 nm/s, and *P*_GABAAR_ = 9 nm/s. (c) Firing rates, represented as quartiles, of all the GCs plotted for the different networks (heterogeneous vs identical input case) they resided in. (d) Cumulative distribution of correlation coefficients of firing rates computed from granule cell firing rates in networks constructed with different forms of age‐related heterogeneities (fully immature, fully mature and variable age). Panels on the top and bottom respectively correspond to networks receiving identical and heterogeneous external inputs from the EC. All three populations were endowed with intrinsic and synaptic heterogeneities. (e) Statistical (mean ± *SEM*) comparison of correlation coefficients obtained with networks endowed with distinct forms of heterogeneities, receiving identical (solid boxes; derived from panel d, top) versus heterogeneous (open boxes; derived from panel d*,* bottom) external inputs. (f) Response (output) correlation plotted as a function of input correlation for identical and heterogeneous external inputs [Color figure can be viewed at wileyonlinelibrary.com]

Our analyses thus far involved a sparse set of 10 (5 from MEC and 5 from LEC) *active* synapses afferent on to the network. As it is established that the number of afferent inputs and associated heterogeneities could alter response decorrelation in different networks (Cayco‐Gajic, Clopath, & Silver, 2017; Li, Aimone, Xu, Callaway, & Gage, 2012), would our conclusions differ if we increase the number of active afferent synapses from the EC? To address this, we repeated our analyses in Figures 6, 7, 8, 9, 10 after doubling the number of active afferent synapses to 20 (10 from MEC and 10 from LEC). The firing rate response profiles of 5 typical cells receiving identical (Figure 11a, top) or heterogeneous (Figure 11a, bottom) inputs are depicted. Similar to prior observations (Figure 6a), it may be noted with identical inputs that the overall firing in different cells are scaled versions of the other cells owing to the identical nature of afferent inputs that reach them (Figure 11a, top). We assessed the correlation values from (Figure 11b,c) and input–output decorrelation in (Figure 11d) networks with identical and heterogeneous afferent inputs. We found our conclusions about channel decorrelation—on the relative dominance of the different local heterogeneities with identical afferent inputs, and on the dominance of afferent heterogeneities over these local heterogeneities when they coexpress—to hold with a scenario where there were more afferent inputs arriving into the network. As a next step in our sensitivity analyses, we asked if our conclusions on the role of different forms of heterogeneities were scalable and were invariant to network size? To test this, we repeated our analyses in Figures 6, 7, 8, 9, 10 with a smaller network made of 100 GCs and 15 BCs, and found our conclusions to scale across different network sizes (Figure 12).

**Figure 13:**
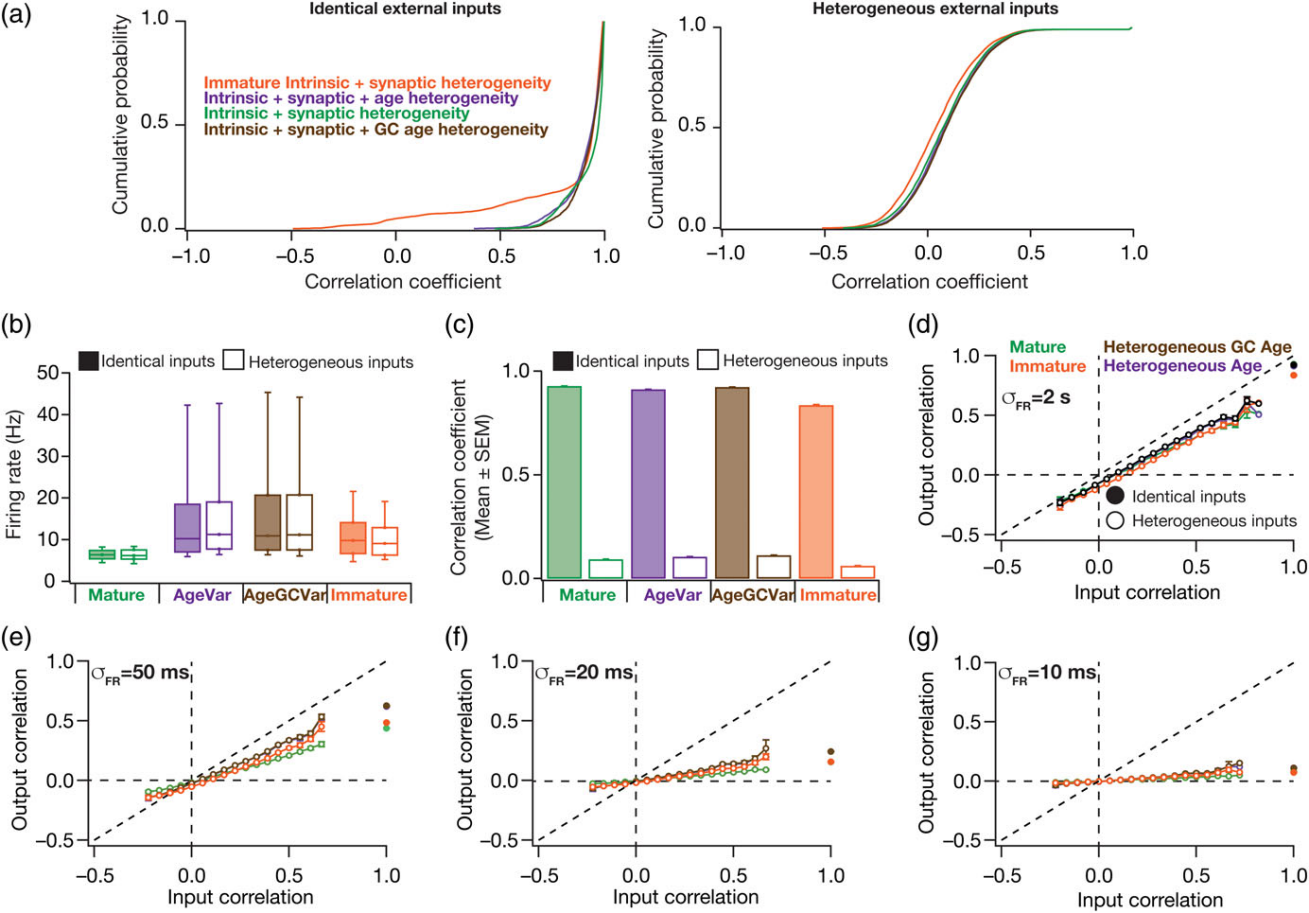
Channel decorrelation in a network receiving heterogeneous external input as a function of neuronal diameter and dependence of input–output correlation on the specific kernel used to compute instantaneous firing rate. (a) Cumulative distribution of correlation coefficients of firing rates computed from granule cell firing rates in networks comprised of 100 GCs and 15 BCs, constructed with different forms of age‐related heterogeneities: fully immature, fully mature, neurogenesis‐induced structural heterogeneity of both GC and BC, and neurogenesis‐induced structural heterogeneity only in GC. Panels on the left and right respectively correspond to networks receiving *identical* and heterogeneous external inputs from the EC. All four populations were endowed with intrinsic and synaptic heterogeneities. (b) Firing rates, represented as quartiles, of all the GCs plotted for the different networks (heterogeneous input vs identical input case) they resided in. (c) Statistical (mean ± *SEM*) comparison of correlation coefficients obtained with networks endowed with distinct forms of heterogeneities, receiving identical (solid boxes; derived from Panel a, left) versus variable (open boxes; derived from Panel a, right) external inputs. (d) Response (output) correlation plotted as a function of input correlation for identical and heterogeneous external inputs. (e–g) Response (output) correlation plotted as a function of input correlation. Shown are three different plots with the firing rate response correlations computed with different values of *σ*_FR_, the standard deviation of the Gaussian kernel used to convert spike trains to instantaneous firing rates (Supporting Information, Figure S1) [Color figure can be viewed at wileyonlinelibrary.com]

There is a growing body of evidence that suggests that the high excitability of immature GCs in the DG is counterbalanced by lower synaptic drive (Dieni et al., 2013; Dieni et al., 2016; Li et al., 2017; Mongiat et al., 2009). To accommodate this into our model and test the impact of such counterbalance on our conclusions, we rescaled the synaptic drive to immature neurons in an excitability‐dependent manner such that the variability in firing rate was reduced (Figure 13b; cf. Figures 10a and 12c). In addition, as lines of evidence for adult neurogenesis in BCs are not as broad as that for GCs, we asked if our conclusions on the dominant role of afferent heterogeneities would hold if adult neurogenesis were restricted only to GCs with the BC population remaining mature. When we repeated our analyses with several distinct configurations involving rescaled inputs and with structural heterogeneities associated with adult neurogenesis confined only to GCs, we found the dominance of afferent heterogeneities and the relative dominance among local heterogeneities when presented with identical afferent stimuli to hold even under these conditions (Figure 13). In addition, we also found that the higher levels of input–output decorrelation that was observed in the purely immature populations receiving heterogeneous inputs (Figures 10c,d, 11d, and 12f) was not observed when this population received rescaled synaptic drive (Figure 13d). This suggested that the apparent increase in the specific levels of decorrelation that was observed earlier was a mere reflection of the huge variability in the firing rates. When this firing rate variability was abolished by the rescaled drive, the amount of decorrelation obtained with heterogeneous afferent inputs matched with that of the other populations (Figure 13d), while still retaining distinctions in correlation coefficients when the inputs were identical (Figure 13a,c).

As a final step in our sensitivity analysis, for all these distinct populations tested here, we asked if our conclusions were dependent on the choice of *σ*_FR_, the standard deviation of the Gaussian kernel that was used in converting spike trains to firing rates. This was essential because the choice of *σ*_FR_ played a critical role in determining the exact value of correlation coefficient computed between two spike trains, with the correlation coefficient increasing with increase in *σ*_FR_ (Supporting Information, Figure S1). Although the specific levels of the decorrelation achieved were higher with lower values of *σ*_FR_, we found that our overall conclusions about the dominance of afferent heterogeneities in mediating channel decorrelation when they coexpress with local heterogeneities held despite changes in *σ*_FR_ (Figure 13d–g). Together, these sensitivity analyses revealed the robustness of our conclusions on both counts: with reference to the relative dominance of local heterogeneities when inputs were identical, and on the significant suppression of the contributions of local heterogeneities to channel decorrelation when they are coexpressed with afferent heterogeneities.

In summary, our results demonstrate that local heterogeneities in intrinsic, synaptic, and neuronal structural (driven by adult neurogenesis) properties contributed to significant levels of channel decorrelation in the presence of *identical* afferent synaptic drive. However, when the network received heterogeneous external inputs, the impact of local heterogeneities on channel decorrelation was strongly suppressed by the dominant role of afferent heterogeneities in mediating neuronal response decorrelation. To elaborate, a central and implicit assumption in the use of an artificial scenario involving identical inputs is that if local network heterogeneities can elicit significant response decorrelation *even* for identical inputs, the contribution of these local network heterogeneities would be sustained or amplified when the inputs become heterogeneous. In other words, the implicit assumption is that there would be significant contributions from local network heterogeneities even under more realistic conditions where the inputs are unique, sparse, and orthogonal (Li et al., 2017). The conclusions of this study instead demonstrate that the contributions of local network heterogeneities are significantly suppressed (not enhanced or sustained) when an additional and physiologically critical (Li et al., 2017) layer of afferent heterogeneities coexpresses in the network.

## 4 DISCUSSION

Adult neurogenesis in the DG has been shown to drive unique, sparse, and orthogonal afferent inputs onto DG neurons (Li et al., 2017), which are postulated to subserve efficacious information transfer by reducing neuronal response correlations (Chow et al., 2012; Padmanabhan & Urban, 2010; Pitkow & Meister, 2012; Tetzlaff et al., 2012; Wiechert et al., 2010). Several forms of local‐network heterogeneities have been implicated in mediating such response decorrelation. However, the orthogonal afferent inputs unique to the DG demand a delineation of the relative contributions of disparate local heterogeneities when they coexpress along with such afferent heterogeneities. An essential requirement for such systematic delineation is a population of heterogeneous conductance‐based neuronal network models, with each network model endowed with disparate sets of heterogeneities. In this study, assembling and assessing such populations of networks, we unveil a novel convergence of cellular‐ and network‐scale degeneracy and a hierarchy of heterogeneities in mediating DG channel decorrelation, with afferent heterogeneities suppressing the contributions of all coexpressing local heterogeneities. Specifically, our experimental design enabled quantitative demonstration of a hierarchy of heterogeneities—synaptic, intrinsic, neurogenesis‐induced structural, afferent connectivity‐induced, in increasing order of dominance when they coexpress—in effectuating channel decorrelation in the DG.

The principal conclusions of the systematic multi‐scale analyses presented here span the cellular and network scales. First, our analyses demonstrate cellular‐scale degeneracy in dentate gyrus neurons, demonstrating independently for granule and basket cells that disparate combinations of passive and active parameters could result in similar signature electrophysiological properties (Figures 3, 4, 5). These conclusions present significant degrees of freedom to these cells, in terms of channel expression profiles, and in the robust maintenance of intrinsic properties and their homeostasis (Hanus & Schuman, 2013; Mittal & Narayanan, 2018; Rathour & Narayanan, 2012; Rathour & Narayanan, 2014). Second, with reference to networks of neurons that received identical inputs, our analyses showed that heterogeneities in intrinsic neuronal properties and local synaptic heterogeneities (including local synaptic inhibition) could drive response decorrelation across neurons, either individually or synergistically when they are expressed together. These analyses also presented a hierarchy of local heterogeneities in mediating response decorrelation, whereby intrinsic heterogeneities were the dominant form between intrinsic and synaptic heterogeneities. In addition, we demonstrated that neurogenesis‐induced structural heterogeneities further enhance the ability of the network to perform input discriminability (Figures 6, 7, 8).

Third, these results also emphasize a potential role for changes in intrinsic neuronal properties as a putative mechanism for rate remapping in granule cells, whereby the rate of firing at a given place field location could be significantly modulated by changes in the intrinsic excitability of the cell, even when the afferent inputs remained the same (Figure 6a). Fourth, incorporating afferent heterogeneities to reflect the specific connectivity pattern and the active recruitment of heterogeneous afferents by the DG network (Andersen, Morris, Amaral, Bliss, & O'Keefe, 2006; Li et al., 2017), we found that the quantitative contributions of local heterogeneities to the emergence of channel decorrelation significantly diminished in the presence of afferent heterogeneities (Figures 10, 11, 12). These results imply that with reference to the dentate gyrus endowed with the expression of afferent heterogeneities and sparse connectivity, analyses on response decorrelation should not merely rely on extrapolations from conclusions derived from scenarios with identical inputs.

Finally and importantly, our results provide a clear case that the interactions among distinct forms of heterogeneities together drive channel decorrelation, with the relative contribution of any form of heterogeneity critically reliant on the expression and the magnitude of other forms of heterogeneities. Akin to several degrees of freedom provided by cellular‐scale degeneracy, with reference to cellular properties and channel combinations, this provides significant degrees of freedom to the DG network in eliciting similar levels of channel decorrelation through the recruitment of several disparate forms of heterogeneities, either independently or together. This novel convergence of cellular‐ and network‐scale degeneracy also suggests degeneracy as an overall framework that can concomitantly accomplish encoding‐related tasks (in our case, the emergence of channel decorrelation) and the maintenance of homeostasis in neuronal response properties (in our case, the intrinsic electrophysiological signatures and firing properties), without any cross‐interferences.

### 4.1 Dominance of input‐driven heterogeneity and implications for the physiological roles for adult neurogenesis

Our results quantitatively demonstrate a dominant role for afferent heterogeneities, driven specifically by the unique network structure of the DG, in driving response decorrelation in the DG. Within our framework, this dominant heterogeneity associated with unique, sparse and orthogonal feed‐forward afferents connecting to each GC (Li et al., 2017), synergistically coupled to the heterogeneous intrinsic properties (including those introduced by adult neurogenesis) and the sparse GC activity that is sharpened by the local inhibitory network, places the DG network as an ideal decorrelating system.

If adult neurogenesis‐induced structural heterogeneities (and associated heterogeneities in excitability) of neuronal properties were not the dominant contributor to channel decorrelation, what could be the precise role of adult neurogenesis in channel decorrelation? One possibility within our framework is that adult neurogenesis could be a mechanism for implementing afferent heterogeneities across DG neurons, whereby new neurons establish connections to afferent fibers in an activity‐dependent manner (Alvarez et al., 2016; Dupret et al., 2007; Marin‐Burgin et al., 2012; Tashiro, Sandler, Toni, Zhao, & Gage, 2006), thereby assigning a specific set of active afferent inputs to new neurons of the same time of birth (Aimone et al., 2006; Aimone et al., 2009; Aimone et al., 2014; Li et al., 2017). In such a scenario, the afferent heterogeneities would be driven by active assignment of spatial connectivity from the EC to individual DG neurons, whereby the novel contexts encountered by the animal are encoded by the temporal onset of neurons. Such active assignments could be driven by activity‐dependent connectivity aided by the hyperplastic, hyperexcitable nature of new neurons, and the resultant afferent heterogeneities (different neurons get different EC inputs) then plays specific roles in response decorrelation, in encoding temporal context and in controlling memory resolution (Aimone et al., 2006; Aimone et al., 2009; Aimone et al., 2014; Alvarez et al., 2016; Dupret et al., 2007; Kropff et al., 2015; Li et al., 2017; Marin‐Burgin et al., 2012; Schmidt‐Hieber et al., 2004; Tashiro et al., 2006). In addition to this, our results suggest that the variability introduced by new neurons in terms of their intrinsic excitability (Figure 8) and in terms of altered excitation–inhibition balance (Figure 7) could also be candidate mechanisms through which adult neurogenesis enhances (beyond what is driven by the specific extent of afferent heterogeneities) the degree of response decorrelation achieved in the DG network (Aimone et al., 2006; Aimone et al., 2009; Aimone et al., 2011; Aimone et al., 2014; Kropff et al., 2015; Severa et al., 2017). In light of this possibility (Li et al., 2017) where neurogenesis mediates the dominant afferent heterogeneities that could be the principal driving force for effectuating response decorrelation, it is important that future studies focus on the extent of heterogeneities in afferent connectivity and synergistic interactions of afferent heterogeneities with local heterogeneities in effectuating input discriminability (Cayco‐Gajic et al., 2017).

### 4.2 Multiscale degeneracy: Convergence of different scales of degeneracy to achieve single‐neuron homeostasis and channel decorrelation

A central premise of robustness in biological function is degeneracy, where distinct structural components could combine to elicit analogous function. Given the several possible routes through which similar function can be achieved, it is possible for biological systems to invoke disparate mechanisms to achieve the same function through very different parametric combinations (Edelman & Gally, 2001; Foster et al., 1993; Goldman et al., 2001; Prinz et al., 2004; Rathour & Narayanan, 2014; Rathour & Narayanan, 2017). In systems that are responsible for encoding of novel information, robust homeostasis of output constitutes only one side of the overall physiological goals (Rathour & Narayanan, 2017). The other side constitutes encoding of new information, which by definition involves changes to certain output characteristics to reflect this encoding process. With specific reference to the DG, an important *encoding* function has been postulated to be response decorrelation, it is important that the focus is not on mere maintenance of robust outputs. If channel decorrelation is considered as the specific function and different classes of heterogeneity are considered as disparate structural components, our conclusions argue for a case where similar degrees of channel decorrelation could be achieved through disparate classes of heterogeneity. These observations are consistent with the overall framework of degeneracy where distinct structural components could come together to elicit analogous function. Thus, there are several layers of degeneracy, spanning the molecular, cellular, network, and behavioral scales of analyses, embedded in the results presented in this study. At the cellular scale, distinct combinations of intrinsic parameters (involving molecular heterogeneity of ion channel properties) come together to elicit analogous cellular response properties. At the network scale, distinct combinations of intrinsic and synaptic properties (with different extents of associated heterogeneities) interact to elicit similar levels of channel decorrelation. Together, our results unveil a systematic convergence of degeneracy spanning different scales of analysis in the DG network, achieving the twin goals of the DG network (channel decorrelation at the network scale and intrinsic homeostasis at the cellular scale) within the broad framework of degeneracy.

The olfactory bulb (OB) is another brain region that expresses adult neurogenesis and has been postulated to play a critical role in channel decorrelation (Chow et al., 2012; Lledo & Valley, 2016; Padmanabhan & Urban, 2010; Wiechert et al., 2010). Although there are similarities in our conclusions with those in the olfactory literature in terms of the roles of neuronal nonlinearites, intrinsic heterogeneities, and inhibition in effectuating channel decorrelation in the *absence* of afferent heterogeneities, the significant distinction in our conclusions is with reference to the dominant role of afferent heterogeneities. We argue that the dominance of afferent heterogeneities is a distinctive feature of the DG circuit, and is reflective of the unique afferent connectivity to the dentate gyrus and the several stark contrasts between the roles of adult neurogenesis in the DG versus the OB circuit (Aimone et al., 2009; Aimone et al., 2010; Aimone et al., 2011; Aimone et al., 2014; Anderson et al., 2007; Chow et al., 2012; Deng et al., 2010; Imayoshi et al., 2008; Kropff et al., 2015; Leutgeb et al., 2007; Li et al., 2017; Lledo & Valley, 2016; Marr, 1971; Padmanabhan & Urban, 2010; Renno‐Costa et al., 2010; Treves & Rolls, 1994; Wiechert et al., 2010; Yassa & Stark, 2011). These well‐established distinctions between the two networks, in conjunction with our conclusions on the unique role of afferent heterogeneities in the DG network suggest that the mechanisms behind achieving decorrelation in the OB and the DG networks are very different. To elaborate, decorrelation in the OB has been postulated to be aided by new laterally inhibiting neurons forming dendrodendritic synapses across *the local circuit* (Chow et al., 2012; Lledo & Valley, 2016; Padmanabhan & Urban, 2010; Wiechert et al., 2010). In contrast, our conclusions here, derived from the specifics of the DG network especially in terms of the role of adult neurogenesis defining *sparse and orthogonal afferent connectivity* (Li et al., 2017), present a dominant role for the afferent heterogeneities supported by synergistic interactions with local heterogeneities. Together, these disparate structural routes to achieve decorrelation further emphasize our conclusions on degeneracy in encoding mechanisms. Apart from the possibility of how distinct forms of heterogeneities could be recruited to achieve analogous levels of decorrelation, the distinctions between the OB and the DG also point to the possibility that the degree of degeneracy could be much broader where the OB and DG could be using adult neurogenesis in very different ways toward achieving response decorrelation.

Finally, this computational study further strengthens the need for engaging (and explicitly modeling) different components of a physiological system, involving emergent properties and degeneracy at each scale, to effectively address questions that require synergistic interactions between components at multiple scales. This study also emphasizes the need to individually account for the disparate biological heterogeneities (and nontrivial interactions among them), that are ubiquitously prevalent in neuronal systems, in assessing physiological processes (Anirudhan & Narayanan, 2015; Das, Rathour, & Narayanan, 2017; Goldman et al., 2001; Marder, 2011; Marder & Goaillard, 2006; Marder & Taylor, 2011; Mittal & Narayanan, 2018; Mukunda & Narayanan, 2017; Prinz et al., 2004; Rathour & Narayanan, 2012; Rathour & Narayanan, 2014; Rathour & Narayanan, 2017; Srikanth & Narayanan, 2015).

### 4.3 Limitations of the analyses and future directions

Although we had systematically incorporated and assessed the role of several forms of heterogeneities into our conductance‐based network models, the analyses presented here have limitations, several of which have their origins traceable to the computational complexity associated with putting together a heterogeneous conductance‐based network where neurons were endowed with several ion channels. From the perspective of sparse *active* connectivity that is observed in decorrelating circuits, we had used lesser number of synaptic inputs. Although this limitation was partly rectified by our simulations with more number of *active* inputs (Figure 11), future studies could theoretically and experimentally assess the impact of sparseness and heterogeneities in number of synapses towards achieving input discriminability (Cayco‐Gajic et al., 2017; Li et al., 2012). In addition, although we did specific analyses addressing the question on scalability (with reference to network size) of our conclusions (Figure 12), it was on a network that was smaller in size. Future studies could extend our analyses by systematically incorporating the several heterogeneities used here into larger networks (Dyhrfjeld‐Johnsen et al., 2007; Morgan, Santhakumar, & Soltesz, 2007; Schneider, Bezaire, & Soltesz, 2012) and assessing if the conclusions are scalable.

We had not modeled or incorporated other cell types within the dentate gyrus into our network model. These other cells include the mossy cells with their unique ability to mediate feedback projections from the CA3, the molecular layer perforant path‐associated (MOPP) cells and other interneurons that are prevalent within the DG (Amaral, Scharfman, & Lavenex, 2007; Li et al., 2012; Scharfman & Myers, 2012). It would be of interest for future studies to ask if the afferent heterogeneities are still dominant even if the other cells and associated local heterogeneities express within the dentate circuit. We believe that our conclusions on the dominance of afferent heterogeneities would still hold because of the several lines of sensitivity analyses presented here, and because the incorporation of afferent heterogeneities was into GC cells, the primary cell type of the dentate gyrus, and is based on strong experimental and theoretical lines of evidence (Aimone et al., 2006; Aimone et al., 2009; Aimone et al., 2014; Li et al., 2017). Furthermore, in this study, we generated immature model cells by altering only the structural parameters with constraints on input resistance as a physiological measurement. Future computational studies could employ stochastic search strategies specific to precise morphological reconstructions of immature neurons (Beining et al., 2017a; Beining, Mongiat, Schwarzacher, Cuntz, & Jedlicka, 2017b), coupled with rigorous electrophysiological characterization of their channels, to incorporate age heterogeneity into model populations.

Although our focus in this study was on channel decorrelation (Figure 1a) in the dentate gyrus, future studies could assess the impact of the disparate set of heterogeneities analyzed here on pattern decorrelation (Figure 1b). Specifically, whereas channel decorrelation deals with reducing redundancy across output profiles of individual channels (neurons), pattern decorrelation enables neuronal representations of input patterns to be more distinct (Figure 1b), thereby allowing efficient classification of input patterns (Wiechert et al., 2010). These studies could involve distinct arenas where the animal traverses, and assess the impact of morphed arenas presented to neuronal structures in the model (Leutgeb et al., 2007; Renno‐Costa et al., 2010). Additionally, conductance‐based models with realistic biophysical models of ion channels provide the ability to assess the impact of distinct ion channels on pattern and channel decorrelations. Future computational studies could focus on the specific contribution of different channels to pattern and channel decorrelations within the framework of degeneracy presented here employing the virtual knockout framework (Anirudhan & Narayanan, 2015; Mittal & Narayanan, 2018; Mukunda & Narayanan, 2017; Rathour & Narayanan, 2012; Rathour & Narayanan, 2014), and test predictions from these simulations using pharmacological agents in electrophysiological and behavioral experiments.

In this study, simplified single compartmental models for both GCs and BCs are used to avoid computational complexities associated with networks of morphologically realistic models. However, given the critical role of DG dendritic structures in input integration and discriminability (Chavlis, Petrantonakis, & Poirazi, 2017; Krueppel et al., 2011; Schmidt‐Hieber et al., 2007), it is essential to expand our analyses to morphologically realistic conductance‐based DG model with differential spatial distributions of MEC and LEC inputs. Such models would also provide an additional layer of morphological heterogeneity (even among mature GC neurons) in dendritic branching patterns. The interactions of the four forms of heterogeneities with the morphological heterogeneity could then be assessed with reference to different forms of response decorrelation in the DG. These analyses, including the role of heterogeneities in dendritic processing in DG neurons in effectuating channel decorrelation or pattern separation (Chavlis et al., 2017) could be assessed using these multi‐compartmental single neuron models that are built with biological dendritic heterogeneities incorporated into them (Rathour & Narayanan, 2014). In this context, a recent study presents an updated model of GC also introducing a toolbox named T2N that is an interface between NEURON, MATLAB and TREES (Beining et al., 2017b). This toolbox—in conjunction with the MPMOSS approach, spanning morphology, and channel distribution of immature and mature neurons—forms an ideal substrate to address these questions in large‐scale network models endowed with morphological heterogeneity as well (Beining et al., 2017b). Finally, our analyses also predict that rate remapping in DG neurons could also be achieved through plasticity of intrinsic excitability (Figure 6a). This could be tested directly by asking questions about whether intrinsic plasticity in the DG could mediate rate remapping, and assessing differences in the expression of intrinsic plasticity in mature versus immature neurons, especially given the well‐established differences in synaptic plasticity profiles between mature and immature neurons.

## CONFLICT OF INTEREST

The authors declare no conflict of interest.

## AUTHOR CONTRIBUTIONS

P.M. and R.N. designed experiments; P.M. performed experiments and carried out data analysis; P.M. and R.N. cowrote the article.

## Supporting information

Figure S1 Demonstration of the dependence of pairwise correlation coefficients on the standard deviation of the Gaussian kernel used to convert neuronal spike trains to instantaneous firing rates. (a) Left: Gaussian kernel used for computing instantaneous firing rates. Middle and right: Instantaneous firing rates computed with the kernel shown on the left for two random spike trains (referred to as Neuron #1 and Neuron #2). It may be noted that increasing the kernel width smoothens the instantaneous firing rates and increases the Pearson's correlation coefficient *R* (*f*_1_, *f*_2_) and Spearman's correlation coefficient *R*s (*f*_1_, *f*_2_), where *f*_1_ and *f*_2_ are the instantaneous firing rate waveforms for Neurons #1 and #2, respectively. (b) Plot showing the increase in *R* (*f*_1_, *f*_2_) and *R*s (*f*_1_, *f*_2_) with increase in σFR
