# Supplementary material for: Disparate forms of heterogeneities and interactions among them drive channel decorrelation in the dentate gyrus: Degeneracy and dominance
Source: Hippocampus. 2018 Dec 7;29(4):378–403. doi: 10.1002/hipo.23035 (PMC6420062; doi:10.1002/hipo.23035)
Supplement: Supplementary file 1 — Figure S1 Demonstration of the dependence of pairwise correlation coefficients on the standard deviation of the Gaussian kernel used to convert neuronal spike trains to instantaneous firing rates. (a) Left: Gaussian kernel used for computing instantaneous firing rates. Middle and right: Instantaneous firing rates computed with the kernel shown on the left for two random spike trains (referred to as Neuron #1 and Neuron #2). It may be noted that increasing the kernel width smoothens the instantaneous firing rates and increases the Pearson's correlation coefficient R (f 1, f 2) and Spearman's correlation coefficient Rs (f 1, f 2), where f 1 and f 2 are the instantaneous firing rate waveforms for Neurons #1 and #2, respectively. (b) Plot showing the increase in R (f 1, f 2) and Rs (f 1, f 2) with increase in σFR [file HIPO-29-378-s001.pdf]

Disparate forms of heterogeneities and interactions among them drive channel decorrelation in the dentate gyrus: Degeneracy and dominance

Poonam Mishra and Rishikesh Narayanan

Cellular Neurophysiology Laboratory, Molecular Biophysics Unit,
Indian Institute of Science, Bangalore, India.

Supporting Information

Figure S1 2

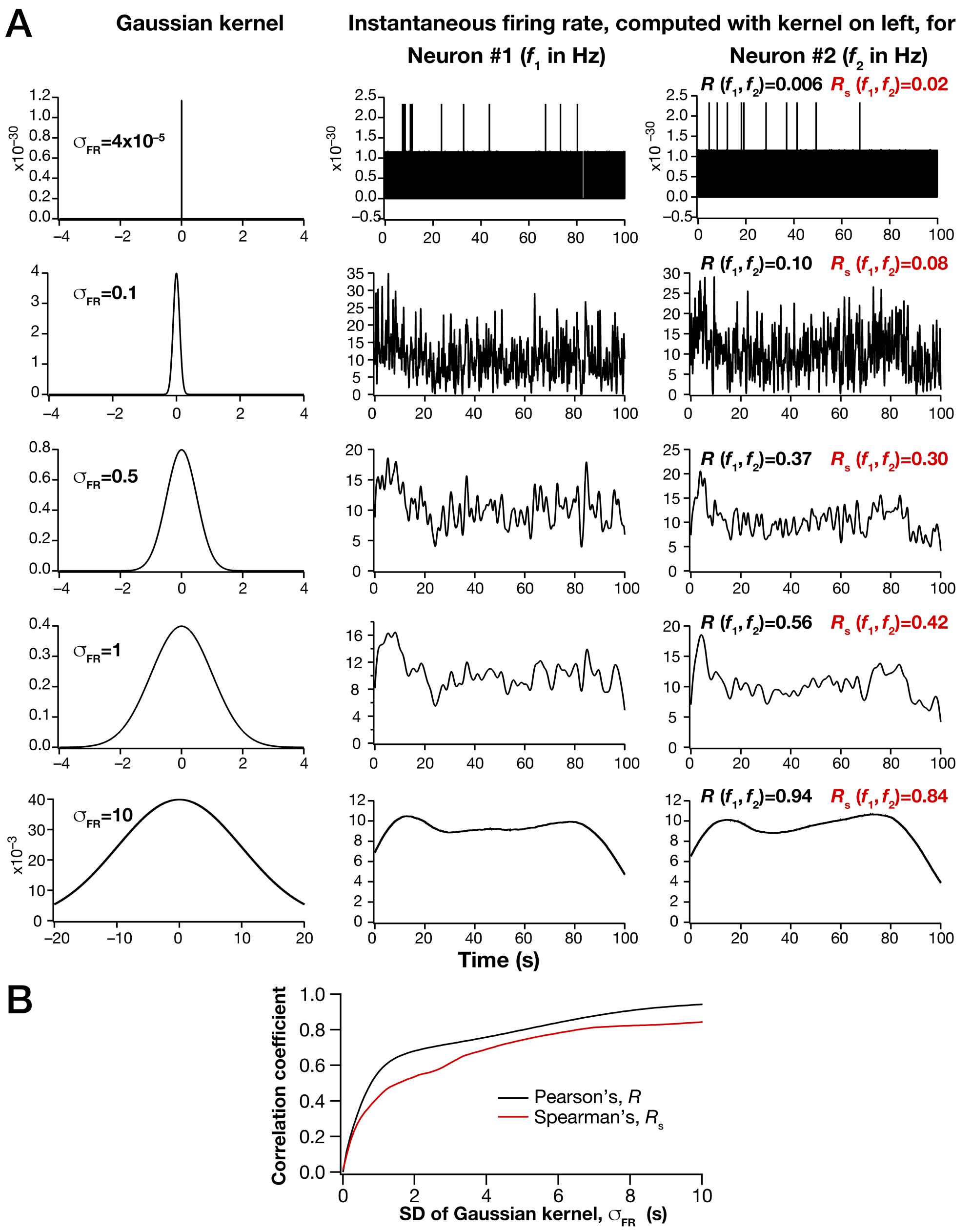

Figure S1. Demonstration of the dependence of pairwise correlation coefficients on the standard deviation of the Gaussian kernel employed to convert neuronal spike trains to instantaneous firing rates. (a) *Left,* Gaussian kernel employed for computing instantaneous firing rates. *Middle and Right,* Instantaneous firing rates computed with the kernel shown on the left for two random spike trains (referred to as Neuron #1 and Neuron #2). It may be noted that increasing the kernel width smoothens the instantaneous firing rates and increases the Pearson's correlation coefficient R (f_1, f_2) and Spearman's correlation coefficient R_s (f_1, f_2), where f_1 and f_2 are the instantaneous firing rate waveforms for neurons #1 and #2, respectively. **(b)** Plot showing the increase in R (f_1, f_2) and R_s (f_1, f_2) with increase in σ_{FR}.
